# Response of Montane Fish Biodiversity to Landscape and Anthropogenic Activity Under Potential Water Quality Pathways

**DOI:** 10.1002/ece3.71279

**Published:** 2025-04-09

**Authors:** Wenjun Zhong, Wanjuan Bi, Yan Zhang, Feilong Li, Zehua Zhang, Xiangyun Huang, Xunjie Liu, Yifan Wang, Song Zhang, Shan Xu, Loïc Pellissier, Xiaowei Zhang

**Affiliations:** ^1^ State Key Laboratory of Water Pollution Control and Green Resource Recycling, School of the Environment Nanjing University Nanjing China; ^2^ Landscape Ecology, Department of Environmental System Science, Institute of Terrestrial Ecosystems ETH Zurich Zurich Switzerland; ^3^ Swiss Federal Research Institute WSL Birmensdorf Switzerland; ^4^ Guangdong Provincial Key Laboratory of Water Quality Improvement and Ecological Restoration for Watersheds, School of Ecology, Environment and Resources Guangdong University of Technology Guangzhou China; ^5^ Key Laboratory of Rivers and Lakes Ecological Health Assessment and Restoration in Yunnan Province, Academician Workstation of Rivers and Lakes Ecological Health Assessment and Restoration in Kunming, Kunming Dianchi Lake Environmental Protection Collaborative Research Center Kunming University Kunming China; ^6^ School of Ecology and Environmental Science Yunnan University Kunming China

**Keywords:** functional diversity, genetic diversity, taxonomic diversity, Yuan River

## Abstract

Mountain river ecosystems, globally recognized biodiversity hotspots shaped by pronounced landscape heterogeneity, are facing intensifying anthropogenic pressures. However, interactions between landscape and anthropogenic activity on montane fish biodiversity remain poorly quantified. Taking the Yuan River (Yunnan, China) as a model system, environmental DNA (eDNA) and partial least squares structural equation modeling (PLS‐SEM) were coupled to disentangle responses of fish biodiversity facets (taxonomic, functional and genetic diversity) to elevation and human footprint gradients. First, eDNA‐derived taxonomic composition (*R* = 0.97 against catch data) demonstrated Cypriniformes and Perciformes dominance. Second, downstream areas exhibited enhanced taxonomic (*R* = 0.32) and functional diversity (*R* = 0.49), contrasting with upstream genetic diversity maxima (*R* = −0.47). Third, elevation gradients and human footprint exerted stronger direct effects on taxonomic diversity than on functional or genetic metrics, independent of spatial autocorrelation. Crucially, PLS‐SEM identified water quality (i.e., total phosphorus (TP), total nitrogen (TN), biochemical oxygen demand (BOD_5_), and total organic carbon (TOC)) as a pivotal mediator linking elevation and human footprint to biodiversity outcomes. Overall, the present study establishes a mechanistic framework for disentangling landscape and anthropogenic drivers of biodiversity change, offering a scalable reference for conservation prioritization in montane freshwater ecosystems.

## Graphical abstract

Patterns and mechanisms of fish taxonomic, functional, and genetic diversity were revealed in a montane river by eDNA. This study provides a new point to explore fish biodiversity facets and their patterns with associated indicators and pathways in montane river ecosystems.

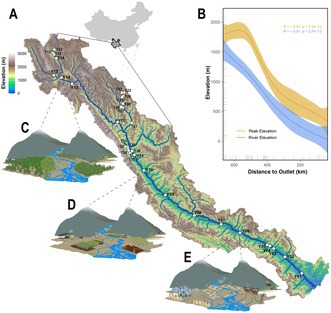

## 1 Introduction

Mountains cover 27% of the Earth's surface and hold more than 80% of the planet's freshwater resources (Romeo et al. 2020; Wang et al. 2022), but are under threat from increasing climate change and anthropogenic activities globally. Montane ecosystems are centred around complex river networks that shape the landscape (Rahbek et al. 2019), connecting mountaintops and lowland plains with a maximum elevations ranging from 300 m to over 4500 m (Sayre et al. 2018). Montane river networks export significant amounts of energy and nutrients, provide habitat or shelter for a wide range of aquatic and riparian organisms, and support migratory corridors (Encalada et al. 2019; Zhu et al. 2023). Eighty percent of amphibians, birds, and mammals currently inhabit montane ecosystems (Romeo et al. 2020). However, the increasing fragmentation of rivers suffering from anthropogenic activities, including landscape changes due to dams and urban development (Best 2019), as well as deteriorating physico‐chemical conditions of water quality (Zhou et al. 2024), is significantly reducing riverine biodiversity and threatening its ecological health (Belletti et al. 2020), even of montane river ecosystems (Bona et al. 2023). Currently, many studies have investigated these impacts on the ecology and biodiversity of lowland rivers below 750 m elevation (Dallaire et al. 2019), but few have focused on the patterns and influences in montane rivers (Encalada et al. 2019).

Fish play an important role in maintaining the biodiversity hotspot, function, and genetic flow of river ecosystems, so fish biodiversity is often considered a factor in biomonitoring and assessment (Su et al. 2021). Fish biodiversity contributes to biomass production and is regulated by trophic networks and nutrient conditions (Zhou et al. 2024), supporting environmental functions and stabilities that shape distinct ecological traits. Compared to plain rivers, increased landscape heterogeneity in montane river networks supports upstream–downstream gradients of fish biodiversity facets (Li et al. 2021; Terui et al. 2021), including taxonomic, functional, and genetic diversity (Pollock et al. 2020). However, montane fish biodiversity facets are either studied independently, using inconsistent methodologies, or are understudied. Thus, efficient approaches are needed for routine biomonitoring of these facets, which cannot be fully and sufficiently understood if based solely on trawling (Mercado‐Silva and Escandón‐Sandoval 2008). Environmental DNA (eDNA), which achieves all genetic materials of fish through a non‐invasive and cost‐effective sampling program even in remote areas with high spatial autocorrelation in river networks (Yao et al. 2022), offers a more precise method of biomonitoring spatial–temporal patterns than conventional surveys (Keck et al. 2022). Recent aquatic surveys have shown that eDNA can successfully reconstruct taxonomic, functional, or genetic information about fishes for entire catchments using only a small volume of water (Cantera et al. 2025; Perry et al. 2024; Yatsuyanagi et al. 2024). Therefore, eDNA is a potentially novel approach in montane fluvial ecosystems as it can be used to rapidly assess distribution patterns of fish biodiversity facets (Figure S1).

Fish biodiversity facets could be changed by both landscape and anthropogenic gradients (Flores‐Galicia et al. 2021), yet their effects and interactions remain unclear in montane rivers. Freshwater ecosystems situated at high elevations or within tributary networks demonstrate a tendency to constrain fish traits, particularly body size and mobility, while concurrently restricting population densities, but also support the occurrence of specific fish sequences in remote areas. This dual ecological function consequently appears to mediate stochastic assembly processes governing fish communities across riverine landscapes characterized by pronounced spatial heterogeneity (Li et al. 2024). Emerging empirical evidence indicates that anthropogenic activities negatively mediate extractive exploitation and species translocations of endemic fish species, which in turn directly determine fish biodiversity facets (Villéger et al. 2011). Moreover, anthropogenic activities can modify the natural landscape by changing land use, altering hydrological regimes, and fragmenting dendritic river networks, which indirectly affect fish biodiversity facets (Su et al. 2021). Thus, it requires a systematic quantification of the interplay between the landscape and anthropogenic variables to which fish biodiversity responds, in particular to understand how these variables mediate these cause‐effect pathways. In addition, even small perturbations of water quality can result in extreme restructuring of fish habitats from upstream to downstream (Li et al. 2022), including eutrophication from phosphorus and nitrogen (Zhou et al. 2024) and higher concentrations of biochemical oxygen demand (BOD_5_) (Hu et al. 2020). However, current monitoring of fish biodiversity facets in montane rivers rarely takes into account the effect patterns under landscape and anthropogenic gradients with the effects of water quality.

**FIGURE 1:**
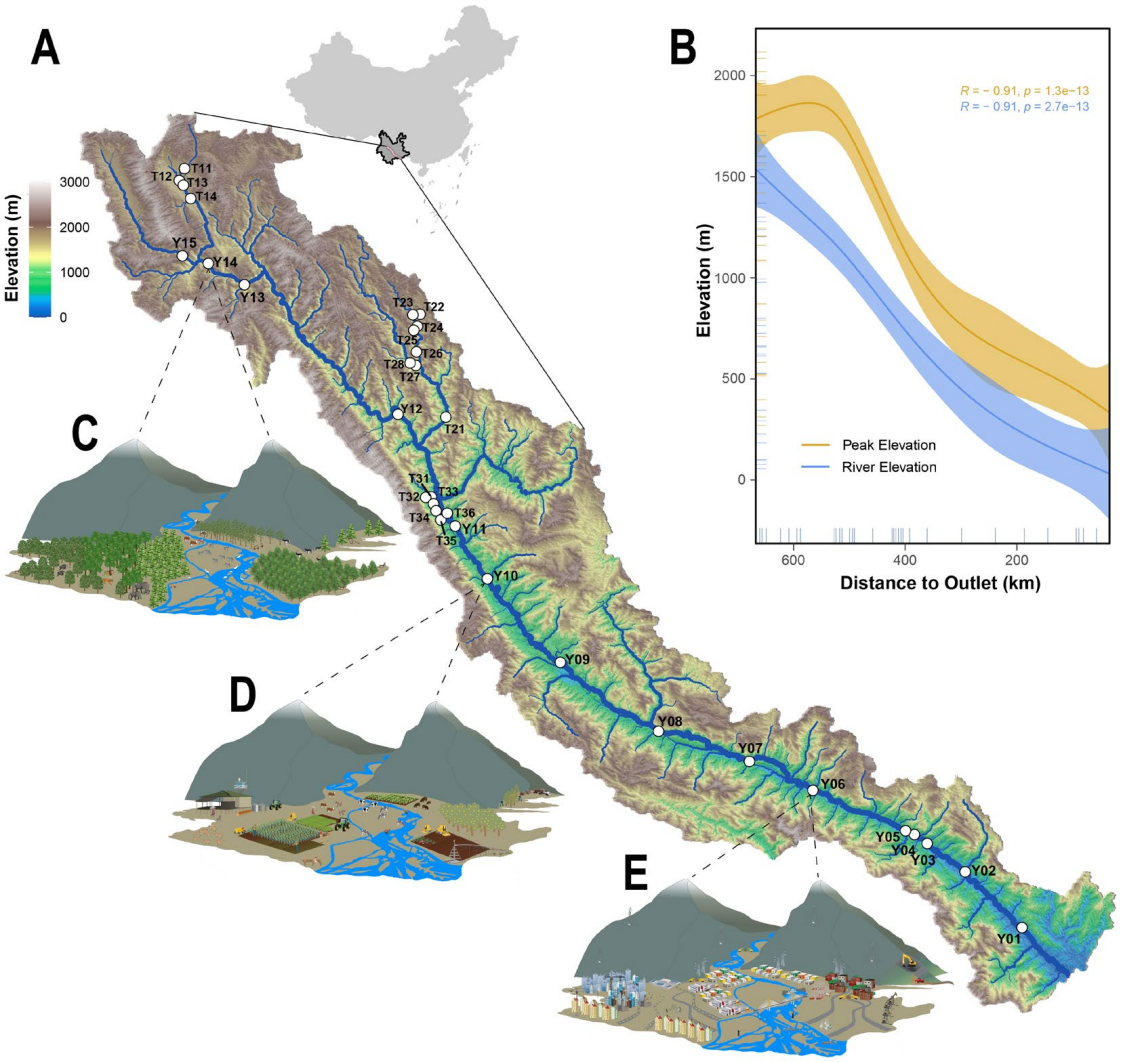
Unique mountainous conditions and environmental characteristics of the Yuan River Basin: (A) topography of the Yuan River Basin and the 33 sampling sites; (B) drop patterns of river valley elevations and mountaintop elevations (fitted by the generalized additive predictive model); examples of the elevation gradient and anthropogenic activity gradient in natural forested areas (C), agricultural areas (D), and urban areas (E).

Here, we investigated the patterns of fish biodiversity facets by eDNA in a typical montane river along landscape and anthropogenic activity gradients, as well as the spatial autocorrelation and tributary effect. The Yuan River (Yunnan, China) traverses near 700 km longitudinally with an elevation gradient exceeding 1000 m, exhibiting pronounced landscape heterogeneity characterized by a 900 m altitudinal disparity between riparian valleys and adjacent montane systems, creating dendritic river networks with diverse habitat characteristics and species richness and supporting the daily lives of almost 200,000 people in cities and towns with agriculture‐dominant industries (Figure 1) (Myers et al. 2000). Taking the Yuan River as a model system, we aimed to: (1) evaluate the effectiveness of eDNA in monitoring fish in montane rivers; (2) analyze the distribution patterns of fish biodiversity facets based on eDNA; (3) model the effects on fish biodiversity facets revealed by eDNA under gradients of elevation and anthropogenic activity, including the effects of spatial autocorrelation and tributaries; and (4) assess the potential pathways from water quality, through elevation and anthropogenic activity, to fish biodiversity. The present study provides a new technical application to explore biodiversity facets of montane river fishes and their patterns associated with environmental factors.

## 2 Materials and Methods

### 2.1 Sampling, Geological Information and Water Quality Recording

Thirty‐three sites were sampled in Yuan River where the lower reaches were dominated by low elevation–low human activity, the middle reaches were dominated by medium elevation–high human activity, and the upper reaches were dominated by high elevation–low human activity (Figure S2). In the tributaries, although these sampling sites were considerably close to each other in the river network, we have demonstrated the rationality of considering these sites independently or together (Text S1). eDNA samples were enriched using three replicate 0.45 μm microporous aqueous filter membranes (JINTENG, China) from 333 mL of water collected evenly from each of the three locations at a sampling site. All filtered samples were buried in dry ice to maintain a low temperature until DNA extraction. To avoid cross‐contamination, disposable nitrile gloves and masks were worn and changed at each site, and sampling bottles were lubricated with in situ water. To model the elevation and water quality pathways and analyze the spatial autocovariate, the geological information including latitude, longitude, and elevation of each sampling site was recorded by GPS logger, and the key water quality parameters including total phosphorus (TP), total nitrogen (TN), 5‐day BOD_5_ and total organic carbon (TOC) were measured by Yunnan Gaoke Environmental Protection Co Ltd. using a multi‐parameter water quality analyzer, which can directly read the above parameters.

### 2.2 Laboratory Procedures

DNA was extracted using the DNeasy Powerwater Kit (Qiagen, Germany) following the standard procedure. DNA from the same site and location was mixed together during the extraction. Fish 12S‐rDNA was amplified using primers named Tele02, which have been shown to have excellent detection efficiency for freshwater fish (Macher et al. 2023; Zhang et al. 2020), while the forward head of the primers was linked by specific 12 bp barcodes. The 25 μL PCR system included 12.5 μL of TB Green Premix Ex Taq II (RR820A, TaKaRa, Japan), 0.5 μL of 10 μM forward and reverse primers, 0.5 μL of ROX Reference Dye (RR820A, TaKaRa), 0.5 μL of BSA solution (PCR Grade) (RFT161, Beijing BioLab Technology Co., China), 1.8 μL of DNA template, and 8.7 μL of DEPC water. The thermocycler running included an initial step of 95°C for 30 s, 31 cycles of 95°C for 30 s' denaturation, 56.3°C for 30 s' annealing, and 72°C for 20 s' extension following a final extension of 72°C for 10 min. Target bands were checked by 2% agarose gel electrophoresis, and all amplified products were mixed equally. Following manufacturer's protocols, VAHTS DNA Clean Beads (N411, Vazyme Biotech Co. Ltd., China) and VAHTS Universal DNA Library Prep Kit for Illumina V3 (Vazyme Biotech Co. Ltd.) were used to construct libraries which were assayed on a Qubit fluorometer (Thermo Fisher Scientific, USA) and sequenced using a PE150 strategy via an Illumina platform (Shanghai Biozeron Biotechnology Co. Ltd., China).

### 2.3 Bioinformatic Analyses

Raw paired‐end sequences were merged by the fastq_mergepairs algorithm from VSEARCH (version 2.14.1) (Rognes et al. 2016). Then, the barcode_splitter script (version 0.18.6) (Leach and Parsons 2019) was used to split the data by sample ID. Demultiplexing and primer trimming were performed on all sequences assembled by VSEARCH in each sample using Cutadapt software (version 3.4) (Martin 2011), with a maximum allowed mismatch error rate of 0.1. Concordant sequences were dereplicated, and the number of times the sequence repeated was counted as reads. All sequences were clustered as amplicon sequence variants (ASVs) through SWARM (version 3.1.0) (Mahé et al. 2014), using a minimum distance of one nucleotide between each sequence (*d* = 1). The “‐‐uchime_denovo” command in VSEARCH (Rognes et al. 2016) was performed to check and remove chimeras. All ASVs were assigned by a lowest common ancestor algorithm ecotag from OBITOOLs (version 1.01.22) (Boyer et al. 2016) with a fish barcode reference database, which was built using all sequences and taxonomic information downloaded from NCBI. Algorithms with obiconvert, ecopcr, obigrep, obiuniq, and obiannotate from OBITOOLs (Boyer et al. 2016) were run in an in silico PCR where the minimum length, maximum length, and maximum mismatches were set as 150, 220, and 3, respectively.

A reference database for native species barcodes was created from the distribution list. Species annotations were prioritized based on 100% similarity and only at the species level. For ASVs not assigned to a species, a secondary annotation was performed using the original barcode reference database, identifying only target sequences with 98% similarity at the species level. Sequences with 96%–98%, 90%–96%, and less than 90% similarity were identified at the genus, family, and order level, respectively. For ASVs having specific names, a cross‐platform and efficient NCBI toolkit named TaxonKit was used to load all taxonomic information. If the order name of an ASV could be found from fishbase.org, the ASV would be judged as fish. All ASVs with less than 10 reads, too short (< 160 bp), too long (> 200 bp), not belonging to fish, or with an abundance frequency of less than 0.001 were deleted to avoid tag‐jump noise (Schnell et al. 2015). The LULU algorithm (Froslev et al. 2017) was then used to clean ASVs identified as erroneous based on sequence identity between ASVs, abundances, and patterns of co‐occurrence. Finally, only curated ASVs in at least two replicates or not detected in any replicates were retained.

### 2.4 Three Biodiversity Facets

First, taxonomic diversity was measured as the number of ASVs (reads > 0) at each site. Secondly, functional diversity was generated from morphological traits of each ASV. Briefly, for ASVs that were annotated to the species level, clear and complete side pictures of adult fish were downloaded from fishbase.org, then 11 original morphological traits (body length, body depth, head depth, caudal peduncle depth, caudal fin depth, eye diameter, eye height, oral gape position, maxillary jaw length, pectoral fin length and pectoral fin position) were measured using ImageJ (http://rsb.info.nih.gov/ij/index.html) (Brosse et al. 2021). These selected morphological traits were combined and calculated as unitless ratios to transform them into nine relative morphological variables related to feeding and locomotor functions (Su et al. 2021; Villéger et al. 2017), which in turn determine their contribution to key ecosystem processes such as the control of food webs and nutrient cycling (Donadi et al. 2017; Mormul et al. 2012) (see details in Text S2). For ASVs that were only annotated to the genus level, traits of the best‐matched species having distribution records in Yunnan were selected. Missing values of traits for the remaining ASVs were interpolated using the Rphylopars package (Goolsby et al. 2017) and combined with evolutionary relationships. Raw data of morphological traits (unitless ratios) of each ASV before and after interpolation were recorded in Tables S1 and S2. All traits were scale‐centered transformed and merged by mFD packages (Magneville et al. 2022) to calculate the functional richness, which measures the convex hull volumes whose vertices were delimited by the species at the edge of multidimensional trait spaces. Thirdly, genetic diversity was based on the fish study (Zhong et al. 2022) by calculating mean nucleotide diversity in each order (see details in Text S2).

### 2.5 River Network Extraction and Mapping the Gradient of Anthropogenic Activity

The river network of Yuan River was extracted through a TauDEM tool (Tarboton 1997) from a 90 m DEM on the Google Earth Engine platform (https://developers.google.com/earth‐engine/datasets/catalog/MERIT_Hydro_v1_0_1) (Yamazaki et al. 2019) using a Yuan River's shapefile generated from HydroBASINS (Lehner and Grill 2013). Applying Pitremove, D8Flowdir and Aread8 methods and setting thresholds of “drainage area” and “maximum reach length” to 200 km^2^ and 1500 m respectively, 1243 reaches were derived with their hydrological information. Then, the moveoutletstostreams algorithm was used to relocate each sampling point to its corresponding segment to avoid deviations in recorded latitude and longitude, and the ID of the stream segment corresponding to each point was obtained. To answer effects from the intensity of anthropogenic activity, an integrated variable that involves eight independent human factors (e.g., pasture, roads, railways, population density, navigable waterways, nighttime light, built environment and cropland), named the human footprint (hereafter footprint) (Mu et al. 2022), was download directly from 10.6084/m9.figshare.16571064 as a raster file. By creating a 1 km buffer around the targeted reach, the average values of the footprint within the buffer were calculated using the mask() function of the terra package (Hijmans 2022). The reason of choosing 1 km buffer was justified in Text S3.

### 2.6 Statistical Analyses

To reveal the consistency with conventional methods compared to eDNA results, historical fish catch records in the Yuan River were compiled from the literature (Chen 2013; Wang et al. 2023) according to whether they had a distribution record in the Yuan River or not, and regressed on the number of ASVs detected by eDNA at the level of each fish order. To demonstrate the effectiveness of eDNA, an ASV‐size and coverage‐based rarefaction and extrapolation sampling curve (a cumulative curve) along with confidence bands was computed and plotted using the iNEXT package (Hsieh et al. 2016). In addition, generalized additive models (GAMs) were used to reveal patterns of biodiversity facets.

To account for the effects of elevation and footprint gradients and to test whether there is an interaction between them, two generalized linear models (GLMs) were constructed for each biodiversity facet: a synergy model (mod1) and a direct model (mod0). The former assumes that elevation and footprint have interactive effects, while the latter assumes that they affect biodiversity facets independently. In addition, because high spatial autocorrelation of eDNA was found in river networks, we complemented the spatial patterns of fish biodiversity with respect to spatial autocorrelation, as well as testing whether such patterns differed due to clustering of tributary sites or differences in the location of tributaries and mainstream by additionally including the variables AutoCor and Type in each GLM.

Briefly, a Moran spatial autocorrelogram was executed in the pgirmess package (Bauman et al. 2018) to obtain the nearest significant spatial autocorrelation distance and then the distance‐weighted autocovariate (i.e., footprint). An autocovariate variable was then constructed for all biodiversity facets using autocov_dist() in the spdep package (Bivand and Piras 2015) to account for spatial dependence and to estimate how each segment reflects the values of neighboring segments. Then we extracted the residuals from the GLM model and used them as an independent spatial variable called AutoCor. Type includes binomial values indicating whether the input sampling site is in the mainstream or not, i.e., 1 indicates mainstream and 0 indicates tributary. To test for interactions between elevation and footprint, the Bayes factor (bf01 = exp.(0.5*(BIC(mod1) − BIC(mod0)))) (Wagenmakers 2007) was used to compare the performance of each model by providing the relative strength of evidence in favor of mod0 over mod1, i.e., there is no interaction when the Bayes factor is less than 1. We also tested the random factor of sampling sites by grouping elevation and footprint. All structures of the above models are detailed in Text S4 and all results are summarized in Tables S3 and S4.

Moreover, to integrate water quality as a potential variable influenced by elevation and footprint gradient (Yang et al. 2023), a partial least squares structural equation modeling (PLS‐SEM) with 100 permutations was performed via the plspm package (Sanchez 2013), using footprint and elevation as formative indicators of the combined four key water quality parameters (see details above), which in turn was also a formative indicator of biodiversity (the latent variable). After assessing the quality of the internal and external models, the path coefficients of each effect were derived. All analyses and plots were conducted in R (version 4.1.2) (R Core Team 2021).

## 3 Results

### 3.1 Biodiversity Discrimination and Resolution by eDNA

**FIGURE 2:**
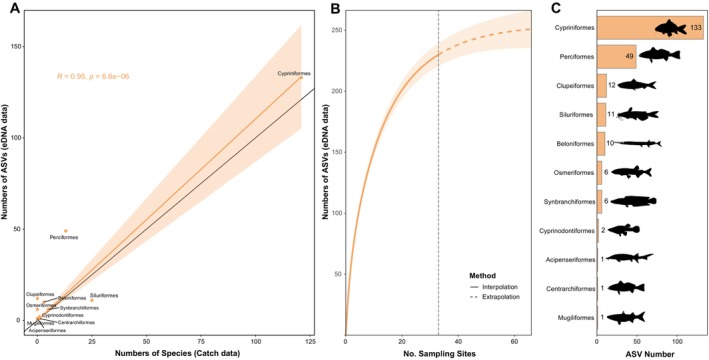
Resolution of fish detection by ASVs in Yuan River: (A) correlation between the number of historical fishing species and ASVs at order level; (B) interpolated and extrapolated cumulative distribution curves with the dashed line indicating the actual number of sampling sites; (C) the number of fish ASVs detected at the order levels.

Fish taxonomic information in the Yuan River obtained by eDNA was highly consistent with occurrence data. A total of 232 fish ASVs were detected, of which 100%, 99.14%, 74.14%, and 44.40% could be annotated to the order, family, genus, and species levels, respectively. The positive correlation between the number of ASVs detected by eDNA at the order level and the number of historical species records is significant (Figure 2A, Pearson *R* = 0.97, *p* < 0.0001). eDNA detected 95.04% of the asymptote of the accumulation curve (244 ASVs), indicating that the samples were relatively representative (Figure 2B). All ASVs belong to 11 orders, where Cypriniformes (133 ASVs) and Perciformes (49 ASVs) were the dominant orders, accounting for 78.45% of the total ASVs (Figure 2C).

### 3.2 Spatial Patterns of Different Biodiversity Facets

**FIGURE 3:**
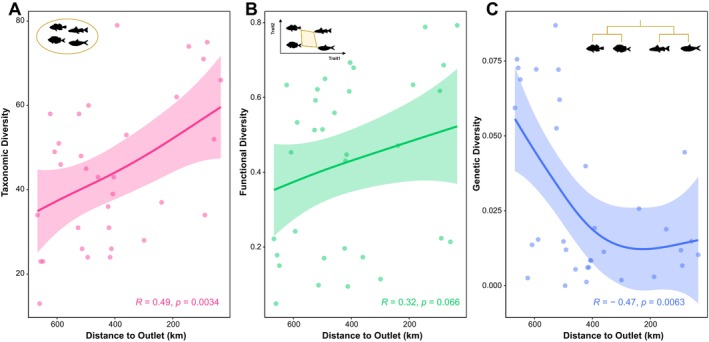
Patterns of taxonomic (A), functional (B), and genetic (C) diversity with distance to the Yuan River outlet. The solid line was simulated by generalized additive model (GAM) with 95% confidence interval.

The downstream sites had higher fish taxonomic and functional diversity, but lower genetic diversity compared to the upstream of Yuan River (Figure 3). Upstream–downstream patterns to the outlet show that approximately monotonically increasing trends were observed in both taxonomic (Figure 3A, GAM: Pearson *R* = 0.49, *p* < 0.0034) and functional diversity (Figure 3B, Pearson *R* = 0.32, *p* < 0.066), while a decreasing and stable trend was observed for genetic diversity (Figure 3C, Pearson *R* = −0.47, *p* < 0.0063). Local values show that taxonomic diversity with an average of 44 fish ASVs varied widely, with T12 near the upstream tributary having the lowest value (13 ASVs) and Y11 near the mainstream having the highest value (79 ASVs). Conversely, T12 exhibited the lowest functional diversity (0.049), whereas the downstream site Y01 had the highest functional diversity (0.793). Furthermore, the first two axes of the functional space PCoA together explained 96.38% of the variation in the corresponding morphological traits, reflecting the different growth, movement, and feeding strategies (Figure S8). In terms of genetic diversity, the upstream site T22 had a maximum value of 0.087.

### 3.3 Effects on Biodiversity Under Elevation and Footprint Gradients

**FIGURE 4:**
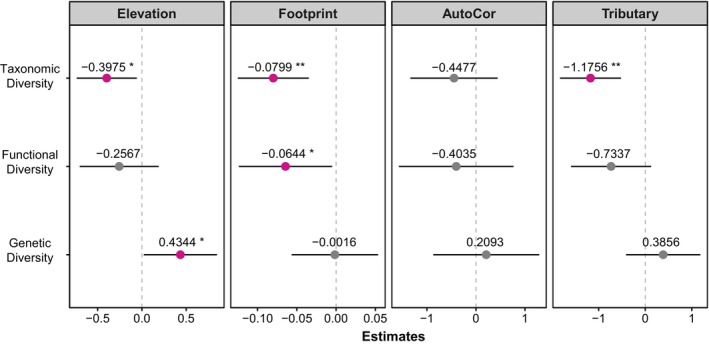
Results of generalized linear models (GLM) testing the effect size of elevation, footprint, spatial autocorrelation and tributary effect. Only significant relationships were colored and marked with asterisks indicating the significance of the corresponding parameter for the corresponding diversity (i.e., *** means *p* < 0.001, ** means *p* < 0.01, * means *p* < 0.05, and no asterisk means *p* 0.05). Note that the elevation, taxonomic diversity, functional diversity and genetic diversity variables were scaled before estimating.

The relationship of each response variable to taxonomic diversity was more significant compared to functional and genetic diversity with no interactions (Figure 4). The effect of elevation gradient was significant only for taxonomic diversity and genetic diversity, which were negatively (Estimates = −0.3975 ± 0.1733) and positively (Estimates = 0.4344 ± 0.2111) correlated, respectively. In contrast, the relationship between footprint and all biodiversity facets was negatively correlated (taxonomic = −0.0799 ± 0.0231; functional = −0.0644 ± 0.0303; genetic = −0.0016 ± 0.0281), although we found no significance in the genetic diversity facet. Notably, the spatial autocorrelation of eDNA in the Yuan River was not significant for any biodiversity facet. The GLM results also suggested that higher taxonomic diversity was more likely to be found in the mainstream compared to tributaries (Estimates = −1.1756 ± 0.3345; Figure S5) but were unable to significantly differentiate between functional and genetic diversity. Remarkably, no interaction between elevation and footprint was observed for any of the fish biodiversity facets (Table S3, Bayes factor (bf01) < 1).

### 3.4 Potential Pathways From Water Quality

**FIGURE 5:**
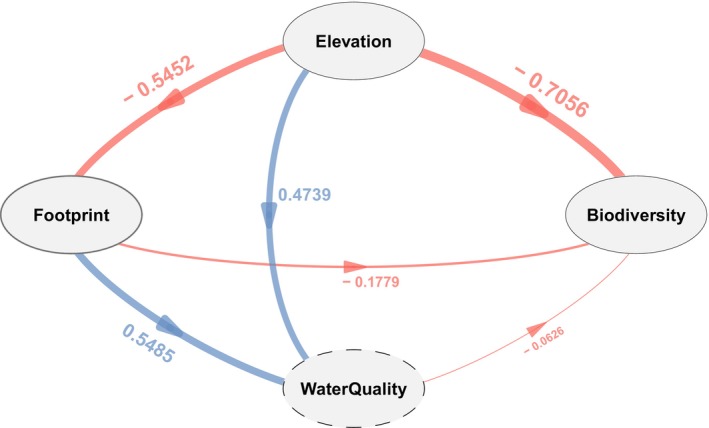
The direct and indirect effect pathways of elevation and footprint on biodiversity and their influence by water quality in the Yuan River calculated by partial least square structural equation modeling (PLS‐SEM). Water quality was expressed as pressure, where the higher the value, the more negative the impact. The arrows indicate the direction of the reflected variables, and the numbers marked with arrows are direct effect values, with negative effects shown in red and positive effects in blue.

Elevation and footprint affected fish biodiversity in Yuan River through direct and indirect pathways through water quality (Figure 5). Directly, elevation gradient had a greater negative effect (effect value = −0.7056) than footprint (effect value = −0.1779) which in turn was negatively affected by elevation (effect value = −0.5452). Indirectly, both elevation and footprint affected fish biodiversity through their potential positive effects on water quality (elevation: effect value = 0.4739; footprint: effect value = 0.5485). Although there were potential effects of elevation and footprint on water quality, the direct effect of water quality was generally small (effect value = −0.0626).

## 4 Discussion

Here, eDNA‐based fish taxonomic, functional, and genetic diversity were measured in Yuan River, a typical dry‐hot valley river basin in China. A strong correlation between the fish monitoring results obtained through eDNA and historical data was observed. Furthermore, downstream areas had higher fish taxonomic and functional diversity, while genetic diversity was lower. The effects of elevation and footprint gradients on fish biodiversity facets were different and not interactive. Both factors were indirectly related to water quality, which in turn affected fish biodiversity facets.

Fish eDNA results in the Yuan River not only matched historical records at the taxonomic level, but also provided valuable genetic information, demonstrating the effectiveness of eDNA application in a montane river. Notably, only 44.40% of ASVs attained species‐level resolution, potentially attributable to suboptimal filtration volumes (333 mL) and primer taxonomic discriminative capacity. Although Tele02 exhibits operational efficiency for freshwater ichthyofauna, its application necessitates rigorous bioinformatic filtering of all detected ASVs, as half of these are non‐target sequences from introduced contaminants, making valid left fish ASVs only 40%–50% of the total (Zhang et al. 2020). Therefore, focusing on species level alone would result in missing more than 50% biodiversity information, indicating that more fish taxonomic diversity could be calculated through ASVs than through species (Skelton et al. 2023). Furthermore, both the ASVs and fish records in the Yuan River (Wang et al. 2023) found the same proportion of dominant fish groups. For instance, Cypriniformes and Perciformes accounted for a total of 79.29% of the physical records, while similarly these two orders also accounted for 78.45% of the fish ASVs revealed by eDNA, demonstrating that not only were Cypriniformes and Perciformes the major taxa, but that the eDNA results of fish ASVs were concordant with traditional methods. The predominance of Cypriniformes and Perciformes also reflects adaptive strategies to montane fluvial landscapes. Cypriniformes thrive in low temperature oligotrophic areas through slow growth rates with long life spans (Zhang et al. 2014), while Perciformes exploit high gradient turbulent flows via rheophilic adaptations, illustrating how hydrological regimes and landscape constraints structure fish assemblages through niche partitioning.

The upstream–downstream patterns of fish taxonomic and functional diversity were similar in Yuan River, while the genetic diversity was opposite. First, fish taxonomic diversity followed a monotonically increasing pattern with proximity to the outlet, which was consistent with established ecogeographic principles where montane remote reaches constraints reduced species richness relative to lowland reaches (McIntosh et al. 2024). As tributaries extending into the mainstream can increase geomorphic richness in downstream areas (de Carvalho Carneiro Mendonça et al. 2021), fish ASVs may mix toward the mainstream, resulting in longer retention times of genetic material and thus more positive detections. Second, fish functional diversity followed a similar and correlative pattern to the taxonomic diversity (Figure S9) with decreasing distance to the outlet. Potentially, the large differences in terrain in the Yuan River lead to variable water temperature and dissolved oxygen environments, which have non‐negligible effects on fish metabolism and growth (Yu et al. 2005). For instance, Bl/Bd, which defines body elongation related to locomotor and nutrient (Toussaint et al. 2016), and CFd/CPd, which defines throttling linked to caudal propulsion efficiency (Webb 1984) have high functional space loading values of 14.56 and 13.89, respectively. These traits will decide the convex hull with specific feeding and growth strategies (Figure S8), including fouling‐tolerant species like *Misgurnus anguillicaudatus*, as well as slender‐bodied fish like *Hyporhamphus intermedius* , and high trophic fish like *Rhinogobius cliffordpopei* . Third, fish genetic diversity was higher in the upper reaches and stabilized in the lower reaches in contrast to the two aforementioned facets. It is hypothesized that catchment‐specific factors at remote upstream, such as mountain range barriers and river network bifurcations, together with significant gradients due to big river slopes, can shape higher abiotic heterogeneity (Schrodt et al. 2019), which creates opportunities for the geographic segregation of different fish genotypes (Menon et al. 2023), leading to more divergent ASVs that can increase genetic diversity. While constructing correlation models by identifying the primary determinants of fish biodiversity facets, as well as their congruence, the new study will help to detail the upstream‐downstream patterns of these facets in the future.

All landscape and anthropogenic variables were found to affect fish biodiversity facets, especially taxonomic diversity. Except for functional diversity, taxonomic and genetic diversity were significantly negatively and positively correlated with elevation gradients, respectively. This dichotomy can be explained by the fact that high water velocities increase dissolved oxygen levels at higher elevation, making montane rivers more resilient to stresses such as pollutants (Sula et al. 2020) and less vulnerable than those at lower elevation, creating a harsher environment that drives fish genotypic specialization while limiting taxonomic diversity (Prunier et al. 2023). Additionally, the consistent mechanism of footprint being negatively correlated with all fish biodiversity facets has been reported previously (Su et al. 2021). It is generally hypothesized that anthropogenic activities may alter the relationship between runoff and watersheds, affecting riparian morphology and sediment content in relation to all biodiversity facets (Allan 2004). Interestingly, insignificant spatial autocorrelations of each biodiversity facet in clustered sites were found, suggesting that eDNA samples can overcome spatial autocorrelation biases associated with classical community assessments by integrating information on biodiversity facets over space (Deiner et al. 2016). Notably, eDNA is not necessarily transported and detected over the same distance for all sites and gradients or consistently over time (Carraro et al. 2020) and will still cluster between tributaries if they are connected. Therefore, although fish communities in the mainstream and tributaries were not significantly grouped in the PCoA analyses (Figure S4), alpha taxonomic diversity between the mainstream and tributaries is still differentiated (Figure 4, Figure S5). Furthermore, there was no significant interaction between elevation and footprint on fish biodiversity (Table S3), indicating that footprint and elevation should be considered as separate explanatory variables when predicting trends in fish biodiversity in this montane area.

Water quality indirectly mediated the effects of elevation and footprint on fish biodiversity in Yuan River. Indirect potential positive effects of water quality were also found with the highest value, which was nine times higher than the direct effect. Footprint and elevation can cumulatively alter water quality and river structures (Leitao et al. 2018) and thus affect fish biodiversity (Allard et al. 2016). For instance, footprint in Yuan River had significantly positive relationships with TOC, which was also co‐correlated to TP and TN (Figure S10); that is, excessive footprint could higher the concentrations of TOC, TP, and TN, upon which water quality deterioration was mainly contingent Water quality was found to be an excellent predictor of biodiversity in the montane river ecosystem (Laura et al. 2016). As shown in the previous study, water quality (e.g., TN) will change closely associated with the intensification of anthropogenic disturbance with the elevation gradients (Li et al. 2022). In the present study, we extended the original limitation of constructing SEM models with fish biodiversity more explicitly, rather than analyzing footprint, elevation, and water quality solitarily. Our modeling results highlighted the necessity of establishing a multidimensional conservation decision model for montane rivers, integrating landscape constraint layers (e.g., elevation gradients), anthropogenic pressure layers (human footprints), and water quality potential layers to protect multi‐facet fish biodiversity. Compared to simple stressor analyses, the multidimensional stressor coupling pathways with fish biodiversity as the response variable provide a comprehensive reference for impact pathways in quantifying ecological thresholds and operationalizing management measures, thereby facilitating the implementation of montane fish conservation initiatives. However, there is still a gap to be filled to determine whether the pathways are applicable to larger or smaller catchments than the Yuan River. Therefore, future studies will be more comprehensive if the joint effects of the above pathways can be tested in relation to catchment area or river complexity.

Some technical parameters are still limited and had to be developed in this study. On the one hand, samples that enriched 333 mL of water per filter membrane may have low biomass and hinder the discovery of rare ASVs or species in the Yuan River, potentially resulting in the loss of valuable biodiversity information, which can be resolved using high‐capacity enrichment (Zhang et al. 2023) or capsule filters (Coutant et al. 2023). On the other hand, montane rivers with large variations in hydrogeological characteristics have a higher resolution of fish habitats (Keck et al. 2022), which can still be affected by environmental changes at a distance through hydrological mediation (Yao et al. 2022). While the characteristics of fish habitats serve as an important reference for conservation planning, they reflect potential links between comprehensive abiotic heterogeneities (i.e., geodiversity) and fish biodiversity that are often overlooked. As geodiversity emerges as a key concept, its geological, geomorphological, hydrological, and pedological facets provide critical insights for studying fish habitat characteristics (Alahuhta et al. 2020). Therefore, through the simultaneous modeling of eDNA and geodiversity, and the synchronous analysis of the distribution mechanism of fish biodiversity facets under combined effects of landscape and footprint variables, more empirical evidence is needed to realize the differentiated conservation of fish biodiversity in different basins from plain rivers to montane rivers.

## 5 Conclusions

Patterns and mechanisms of fish taxonomic, functional, and genetic diversity were revealed in a montane river by eDNA using the case of Yuan River: (1) fish taxonomic information from eDNA was highly consistent with catch data; (2) spatial distributions of fish taxonomic and functional diversity were higher downstream while genetic diversity was higher upstream; (3) with no interaction effects and no spatial autocorrelation, the relationships between elevation, footprint, and the effect of tributary on taxonomic diversity were more significant compared to the other biodiversity facets; and (4) elevation and footprint were found to affect fish biodiversity through direct and indirect pathways via water quality, providing a new point to explore fish biodiversity facets and their patterns with associated indicators and pathways in montane rivers. In this context, establishing a systematic conservation framework that integrates landscape constraints—anthropogenic stressors—water quality responses cascading effects provides a scientific basis for elucidating the maintenance mechanisms of fish biodiversity in montane rivers and formulating adaptive management strategies.

## Author Contributions

**Wenjun Zhong:** conceptualization (equal), data curation (equal), formal analysis (equal), funding acquisition (equal), investigation (equal), methodology (equal), software (equal), visualization (equal), writing – original draft (equal). **Wanjuan Bi:** data curation (equal), methodology (equal), software (equal). **Yan Zhang:** investigation (equal), methodology (equal). **Feilong Li:** investigation (equal), methodology (equal). **Zehua Zhang:** data curation (equal), methodology (equal). **Xiangyun Huang:** methodology (equal), software (equal). **Xunjie Liu:** methodology (equal), software (equal). **Yifan Wang:** methodology (equal). **Song Zhang:** software (equal), visualization (equal). **Shan Xu:** conceptualization (equal), supervision (equal). **Loïc Pellissier:** supervision (equal). **Xiaowei Zhang:** conceptualization (equal), funding acquisition (equal), project administration (equal), supervision (equal), writing – review and editing (equal).

## Conflicts of Interest

The authors declare no conflicts of interest.

## Supporting information


Data S1.

Data S2.

Data S3.

Data S4.

## Data Availability

Raw sequencing data were deposited in the NCBI Bioproject database (accession number: PRJNA1212293). In addition, raw data for ASV of eDNA (0–1 distribution) and genetic diversity results at different taxonomic levels are each provided as a separate file as the Supporting Information.
