# Supplementary material for: Response of Montane Fish Biodiversity to Landscape and Anthropogenic Activity Under Potential Water Quality Pathways
Source: Ecol Evol. 2025 Apr 9;15(4):e71279. doi: 10.1002/ece3.71279 (PMC11981958; doi:10.1002/ece3.71279)
Supplement: Supplementary file 4 — Data S4. [file ECE3-15-e71279-s004.pdf]

Supporting Information

Response of Montane Fish Biodiversity to Landscape and Anthropogenic Activity under Potential Water Quality Pathways

Wenjun Zhong [a, d, e], Wanjuan Bi [a], Yan Zhang [a], Feilong Li [f], Zehua Zhang [a], Xiangyun Huang [a], Xunjie Liu [a], Yifan Wang [a], Song Zhang [a], Shan Xu [c], Loïc Pellissier [d, e], Xiaowei Zhang [a, b ,c] *

[a] State Key Laboratory of Water Pollution Control and Green Resource Recycling, School of the Environment, Nanjing University, 210023 Nanjing, China
[b] School of Ecology and Environmental Science, Yunnan University, Kunming 650500, China
[c] Key Laboratory of Rivers and Lakes Ecological Health Assessment and Restoration in Yunnan Province, Academician Workstation of Rivers and Lakes Ecological Health Assessment and Restoration in Kunming, Kunming Dianchi Lake Environmental Protection Collaborative Research Center, Kunming University, 650214 Kunming, China
[d] Landscape Ecology, Institute of Terrestrial Ecosystems, Department of Environmental System Science, ETH Zurich, 8092 Zurich, Switzerland
[e] Swiss Federal Research Institute WSL, 8903 Birmensdorf, Switzerland
[f] Guangdong Provincial Key Laboratory of Water Quality Improvement and Ecological Restoration for Watersheds, School of Ecology, Environment and Resources, Guangdong University of Technology, 510006 Guangzhou, China

* Corresponding author: Prof. Dr. Xiaowei Zhang
E-mail: zhangxw@ynu.edu.cn

Number of SI Page: 28
Number of SI Text: 4
Number of SI Function: 1
Number of SI Table: 4
Number of SI Figure: 10

Text S1 – Demonstrate effects of clustered sampling sites

In fact, when we designed the sampling sites, we had thought that there were significant differences in human activities and elevation between the mainstem and the different tributaries upstream, so we set up sampling sites in the mainstem and in three geographically different tributaries. For the tributaries, although these sampling sites were very close to each other in the river network on the map, upon arrival we found that these tributary sites that were very close to each other in the river network were reached by long and winding mountain roads, and we thought that the construction of the roads would change the structure of the river channel itself, which would change the natural state of the river and thus might have an effect on the fish community, so we kept these sampling sites design, even though they appeared to be very close together. To allay concerns about the potential impact of tributaries in our model, we added tests of differences in elevation and footprint for both mainstem and tributaries. Although two of the tributaries had regional differences in elevation from the mainstem, footprint did not differ regionally, and even some of the tributaries had footprint values that spanned within the span of the mainstem, with no significant range differences, and only one of the three tributaries had significant elevation and footprint differences from the others (**Figure S3**).

We also analysed fish ASV differences between tributaries and the mainstem by PCoA for the three biodiversity facets (**Figure S4**), and we found that for taxonomic diversity, the community structure of the mainstem overlapped that of the other three tributaries, and although there were some differences in community structure among the three tributaries, there is also some overlap, so the community composition is partially similar between them, which can also be seen in the PCoA result of functional diversity, where it is difficult to distinguish between the fish traits of the mainstem and tributaries. In addition, although there were overlaps between the tributaries, the mainstream and tributaries differed significantly in the composition of ASVs, which may affect the results of our model. Therefore, we included the effect of tributaries on fish biodiversity facet in our GLM model, but for alpha diversity, both statistical models (**Table S3**) and Kruskal-Wallis tests (**Figure S5**) showed that tributaries differed significantly from the mainstem only in the taxonomic diversity facet.

Therefore, all the above results indicate that there are no significant differences whether the clustered sites are considered independently or together.

Text S2 – Details of functional and genetic diversity calculation

1. Measurement of Functional Traits

For functional diversity, 11 original morphological traits including body length (BL), body depth (BD), head depth (HD), caudal peduncle depth (CPD), caudal fin depth (CFD), eye diameter (ED), eye height (EH), oral gape position (MO), maxillary jaw length (JL), pectoral fin length (PFL) and pectoral fin position (PFI) were calculated as nine relative morphological variables, which were body elongation (BL/BD), vertical eye position (EH/BD), relative eye size (ED/HD), mouth position (MO/BD), relative upper jaw length (JL/HD), relative head size (HD/BD), vertical pectoral fin position (PFI/BD), pectoral fin size (PFL/BL), and caudal fin size (CFD/CPD). The 9 selected morphological traits (unitless ratios) are commonly used to assess the functional diversity and are linked to the feeding and locomotor functions, which in turn determine their contribution to key ecosystem processes such as the control of food webs and nutrient cycling.

The original 11 morphological traits were measured through ImageJ as follows:

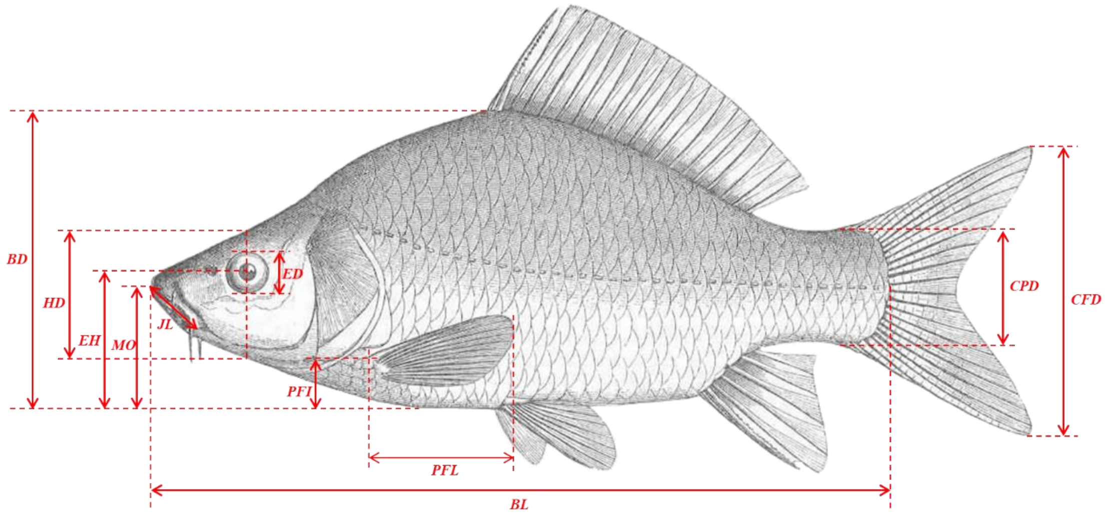

Variable	Definition	Measurement through ImageJ
BL	Body Length	Horizontal length from the most anterior tip of the muzzle to the base of the caudal fin (generally the end of the lateral line)
BD	Body Depth	Vertical distance from the top of the dorsal surface of the fish (except for the dorsal fin) to the baseline of the fish's underside
HD	Head Depth	Perpendicular length of the line passing through the center point of the eye and the two points of intersection with the body of the fish
CPD	Caudal Peduncle Depth	Vertical length of the line between the beginning of the caudal fin and the two points of intersection of the body of the fish

Variable	Definition	Measurement through ImageJ
CFD	Caudal Fin Depth	Maximum extension width of the caudal fin
ED	Eye Diameter	Diameter of one eye past the center point of the eyeball
EH	Eye Height	Vertical distance from the center point of the eye to the baseline of the bottom of the fish body
MO	Oral Gape Position	Vertical distance from the apex of the upper edge of the mouth to the baseline of the bottom of the body of the fish
JL	Maxillary Jaw Length	Length from the tip of the muzzle to the corner of the mouth
PFL	Pectoral Fin Length	Horizontal length of the pectoral fin apex extended to the longest flipper
PFI	Pectoral Fin Position	Vertical distance from the apex of the pectoral fin to the baseline of the bottom of the body of the fish

P.S.: The example fish is a standardized side picture of *Carassioides acuminatus* downloaded from animalia.bio

2. Genetic Diversity

For the classification level of each fish, ASVs that were not assigned to the corresponding level (i.e., order level) and ASVs with only one annotated result (e.g. *Scorpaeniformes* was annotated by only one ASV, so this order would be removed in the analysis of genetic diversity at the order level) were first deleted. Then, MUSCLE3 algorithm was used for multiple sequence alignment of all ASV sequences in the same order in a specific region, and only the pairing ratio of sequence overlap > 50% was retained. The nucleotide diversity (Function 1, i.e. genetic diversity) of fish order was estimated based on the average number of changed base sites of paired sequences of all ASVs in each order. Then, a unique label for each point was made, which was assigned to the species whose genetic diversity was calculated according to whether the ASVs was detected at different points, and the average value of genetic diversity of different orders at different points was calculated.

$$\Pi = \frac{1}{C_n^2}\sum_{i=1}^{n-1}\sum_{j=i+1}^{n}\frac{k_{ij}}{m_{ij}} \quad \text{(Function 1)}$$

where k_{ij} is the number of nucleotides that are different between sequence i and sequence j, n is the number of sequences, C_n^2 is the number of possible pairwise comparisons, and m_{ij} is the number of shared base pairs between sequence i and sequence j.

Because our study chose nucleotide diversity (rather than haplotype diversity) as the metric for calculating genetic diversity, which is essentially a comparison of the average number of nucleotide differences per locus between two sequences randomly selected from the same population, only a certain number of sequences with base differences within the sampling loci can make the calculation of nucleotide diversity mathematically meaningful. Therefore, in the calculation matrix, only a certain number of sequences with base differences within the sampling loci can make the calculation of nucleotide diversity mathematically meaningful. In other words, the more sequences with base differences in the sampled loci, the higher the probability of obtaining a non-zero value in the calculation of nucleotide diversity to make the genetic diversity between the loci comparable.

Due to the small sampling volume of each site, only 44.40% of the total ASVs could be annotated to the species level, which means that more than half of the sequences would be lost if we performed the genetic diversity calculations only at the species level, leading to a potentially biased genetic diversity calculation. For this reason, we provide the raw input data and the output genetic diversity results at the order, family, genus and species levels (see details in another supplementary file). The results of genetic diversity calculations based on different taxonomic levels also showed that when genetic diversity was calculated at the species level, 36.37% of the 33 loci had a genetic diversity of 0. Therefore, we chose to calculate ASVs at the order level in order to capture as many differences between sequences as possible, after all 100% of them were successfully annotated at the order level.

Actually, the results of the linear models showed that genetic diversity would not differ from the calculated results due to the selection of different taxonomic levels, i.e, Genetic diversity at the order level was significantly positively correlated with genetic diversity at the family level (**Figure S6**A, Pearson R = 0.86, $p < 0.001$), genus level (**Figure S6**B, Pearson R = 0.90, $p < 0.001$) and species level (**Figure S6**C, Pearson R = 0.50, $p = 0.0033$), although there were more 0-value effects on genetic diversity at the species level. We therefore retained the results of the order-level genetic diversity analysis in the manuscript.

Text S3 – Justification for the choice of buffer distance

The scale at which environmental factors are considered is an important parameter that can determine the strength of the relationship between fish distribution and predictor variables. In our original trials, buffers was only used to extract the average footprint values of each reach, so the distance of 0.5 km, 1 km, 1.5 km, 2 km, 3 km and 5 km buffers were created to test the difference in footprint values. According to the Wilcoxon signed rank test of the footprint values in each buffer, we found that there were no significant changes from 0.5 km to 3 km buffers, except for the 5 km buffer (**Figure S7**A). However, the shapes of the 2 km and 3 km buffers of each sampling reach had much more overlap than the 0.5 km, 1 km and 1.5 km buffers, so we kept the short distance buffer around the reaches. We also found highly consistent values of the footprint with those of the 1 km buffer area, comparing which values extracted from the 0.5 km buffer were overestimated (**Figure S7**B) while those extracted from the 1.5 km buffer were underestimated (**Figure S7**C), so we ultimately chose the 1 km buffer for the footprint analysis.

Text S4 – Details of generalized linear model (GLM)

In the GLM, two models were executed for each biodiversity facet to account for the effects of elevation and footprint: a synergy model (mod1) and a direct model (mod0). The former assumes interactive effects between elevation and footprint, and the latter assumes independent effects. In order to distinguish and select the better one, we calculated the Bayes factor (below is bf01). The models' structures are as following:

```
mod1 <- glm(Response ~ altitude+footprint+altitude:footprint+Type)
mod0 <- glm(Response ~ altitude+footprint+Type)
bf01 <- exp(0.5*(BIC(mod1) - BIC(mod0)))
```

where Response includes all three biodiversity facets and Type includes binomial values indicating whether the input sampling site is in a mainstream or not, i.e. 1 indicates mainstream and 0 indicates tributary. If the Bayes factor of each biodiversity facet was less than 1, the mod1 was rejected and the parameter of the interaction was marked as n.s. Otherwise, the coefficients for each parameter were labelled with both standard error (SE) and significance (p-value). Consequently, there were no significant interaction found in the results (see details in **Table S3**).

Moreover, considering adding a random factor to our GLM model, we also tested the random factor of sampling sites using function lmer(), where elevation and footprint were grouped as three levels of low (elevation <600 or footprint <15), moderate (600< elevation <1200 or 15< footprint <20) and high (elevaton > 1200 or footprint >20), and formated them as the following:

```
Mod_random1 <- lmer(Response ~ altitude+footprint+Type+ 1|altitude_type)
Mod_random2 <- lmer(Response ~ altitude+footprint+Type+ 1|footprint_type)
```

Then we compared these random model with the direct model (mod0, based on no interactions so we chose mod0) by AIC value, results showed that mod0 was better than the random (**Table S4**), which meant the direct model could explain enough about the patterns and relationships.

Supplementary Tables

Table S1. Original morphological traits (unitless ratios) of each ASV before interpolation. NA means that there are no trait data of this ASV, because it cannot be assigned to at least genus level.

ASVID	BL/BD	EH/BD	ED/HD	MO/BD	JL/HD	HD/BD	PFI/BD	PFL/BL	CFD/CPD
ASV_71	NA	NA	NA	NA	NA	NA	NA	NA	NA
ASV_468	3.212466	0.427381	0.417938	0.577176	0.24111	0.599592	0.221485	0.24947	2.08706
ASV_103	NA	NA	NA	NA	NA	NA	NA	NA	NA
ASV_405	4.825942	0.845115	0.391378	0.708737	0.330631	0.837906	0.579433	0.247546	1.714348
ASV_265	NA	NA	NA	NA	NA	NA	NA	NA	NA
ASV_120	NA	NA	NA	NA	NA	NA	NA	NA	NA
ASV_248	4.542123	0.677769	0.425633	0.432227	0.502071	0.634437	0.279669	0.189343	2.474074
ASV_47	4.179464	0.574374	0.690359	0.651595	0.47403	0.39095	0.307862	0.227394	2.030046
ASV_480	NA	NA	NA	NA	NA	NA	NA	NA	NA
ASV_455	4.825942	0.845115	0.391378	0.708737	0.330631	0.837906	0.579433	0.247546	1.714348
ASV_239	NA	NA	NA	NA	NA	NA	NA	NA	NA
ASV_18	NA	NA	NA	NA	NA	NA	NA	NA	NA
ASV_320	NA	NA	NA	NA	NA	NA	NA	NA	NA
ASV_515	3.866011	0.304009	0.561919	0.308817	0.902564	0.448664	0.212404	0.177473	2.31541
ASV_395	NA	NA	NA	NA	NA	NA	NA	NA	NA
ASV_280	3.570692	0.613874	0.378915	0.562646	0.156111	0.613992	0.376594	0.205325	1.929961
ASV_448	NA	NA	NA	NA	NA	NA	NA	NA	NA
ASV_201	NA	NA	NA	NA	NA	NA	NA	NA	NA
ASV_535	4.506638	0.742256	0.172539	0.544557	0.64476	0.862734	0.79948	0.211437	1.933169
ASV_475	4.698873	0.799085	0.288624	0.446362	0.344732	0.87743	0.753847	0.268948	1.521564
ASV_10	NA	NA	NA	NA	NA	NA	NA	NA	NA
ASV_452	NA	NA	NA	NA	NA	NA	NA	NA	NA
ASV_144	3.570692	0.613874	0.378915	0.562646	0.156111	0.613992	0.376594	0.205325	1.929961
ASV_533	4.506638	0.742256	0.172539	0.544557	0.64476	0.862734	0.79948	0.211437	1.933169

ASVID	BL/BD	EH/BD	ED/HD	MO/BD	JL/HD	HD/BD	PFI/BD	PFL/BL	CFD/CPD
ASV_129	2.815345	0.512375	0.39286	0.529604	0.348707	0.418344	0.314064	0.204297	2.491324
ASV_59	NA	NA	NA	NA	NA	NA	NA	NA	NA
ASV_654	3.212466	0.427381	0.417938	0.577176	0.24111	0.599592	0.221485	0.24947	2.08706
ASV_546	4.200283	0.533449	0.608816	0.644464	0.435943	0.383369	0.388887	0.200773	2.613008
ASV_65	4.551415	0.409171	0.491778	0.425822	0.993017	0.456379	0.284047	0.200356	1.615996
ASV_118	4.506638	0.742256	0.172539	0.544557	0.64476	0.862734	0.79948	0.211437	1.933169
ASV_649	NA	NA	NA	NA	NA	NA	NA	NA	NA
ASV_101	4.506638	0.742256	0.172539	0.544557	0.64476	0.862734	0.79948	0.211437	1.933169
ASV_115	2.858622	0.424135	0.471079	0.448329	0.368205	0.417241	0.231032	0.160434	3.093318
ASV_417	3.710242	0.524258	0.255189	0.362992	0.255635	0.758452	0.209831	0.202143	3.345455
ASV_230	NA	NA	NA	NA	NA	NA	NA	NA	NA
ASV_507	NA	NA	NA	NA	NA	NA	NA	NA	NA
ASV_243	3.512324	0.462105	0.389313	0.462177	0.302473	0.472876	0.220223	0.173139	2.069789
ASV_206	NA	NA	NA	NA	NA	NA	NA	NA	NA
ASV_429	4.200283	0.533449	0.608816	0.644464	0.435943	0.383369	0.388887	0.200773	2.613008
ASV_13	NA	NA	NA	NA	NA	NA	NA	NA	NA
ASV_254	NA	NA	NA	NA	NA	NA	NA	NA	NA
ASV_69	NA	NA	NA	NA	NA	NA	NA	NA	NA
ASV_354	NA	NA	NA	NA	NA	NA	NA	NA	NA
ASV_555	NA	NA	NA	NA	NA	NA	NA	NA	NA
ASV_550	5.624455	0.734304	0.138965	0.491324	0.596095	0.583831	0.398839	0.120422	1.141527
ASV_43	13.42302	0.752358	0.532967	0.495411	4.128165	0.849087	0.472997	0.098307	3.339305
ASV_304	3.512324	0.462105	0.389313	0.462177	0.302473	0.472876	0.220223	0.173139	2.069789
ASV_393	4.716911	0.562914	0.352426	0.251571	0.673874	0.713826	0.160374	0.177559	3.0899
ASV_299	NA	NA	NA	NA	NA	NA	NA	NA	NA
ASV_166	3.570692	0.613874	0.378915	0.562646	0.156111	0.613992	0.376594	0.205325	1.929961
ASV_437	4.698873	0.799085	0.288624	0.446362	0.344732	0.87743	0.753847	0.268948	1.521564
ASV_55	NA	NA	NA	NA	NA	NA	NA	NA	NA
ASV_423	NA	NA	NA	NA	NA	NA	NA	NA	NA
ASV_339	NA	NA	NA	NA	NA	NA	NA	NA	NA

ASVID	BL/BD	EH/BD	ED/HD	MO/BD	JL/HD	HD/BD	PFI/BD	PFL/BL	CFD/CPD
ASV_127	2.204373	0.474314	0.333152	0.427894	0.360825	0.503246	0.255912	0.158555	3.05464
ASV_416	NA	NA	NA	NA	NA	NA	NA	NA	NA
ASV_142	4.825942	0.845115	0.391378	0.708737	0.330631	0.837906	0.579433	0.247546	1.714348
ASV_397	5.167303	0.860773	0.281463	0.491529	0.668039	0.916246	0.670245	0.24011	1.557659
ASV_359	7.587141	0.72812	0.354798	0.597131	1.218244	0.601995	0.238299	0.093549	1.337701
ASV_68	4.642457	0.989068	0.295348	1.017942	0.673488	0.8383	0.675189	0.20749	1.274122
ASV_114	NA	NA	NA	NA	NA	NA	NA	NA	NA
ASV_606	NA	NA	NA	NA	NA	NA	NA	NA	NA
ASV_383	NA	NA	NA	NA	NA	NA	NA	NA	NA
ASV_214	5.443378	0.553784	0.575095	0.552946	0.543931	0.40663	0.639276	0.174314	2.281855
ASV_46	NA	NA	NA	NA	NA	NA	NA	NA	NA
ASV_415	NA	NA	NA	NA	NA	NA	NA	NA	NA
ASV_672	NA	NA	NA	NA	NA	NA	NA	NA	NA
ASV_155	NA	NA	NA	NA	NA	NA	NA	NA	NA
ASV_41	NA	NA	NA	NA	NA	NA	NA	NA	NA
ASV_124	NA	NA	NA	NA	NA	NA	NA	NA	NA
ASV_252	5.624455	0.734304	0.138965	0.491324	0.596095	0.583831	0.398839	0.120422	1.141527
ASV_178	NA	NA	NA	NA	NA	NA	NA	NA	NA
ASV_189	NA	NA	NA	NA	NA	NA	NA	NA	NA
ASV_303	4.200283	0.533449	0.608816	0.644464	0.435943	0.383369	0.388887	0.200773	2.613008
ASV_378	NA	NA	NA	NA	NA	NA	NA	NA	NA
ASV_563	4.716911	0.562914	0.352426	0.251571	0.673874	0.713826	0.160374	0.177559	3.0899
ASV_30	NA	NA	NA	NA	NA	NA	NA	NA	NA
ASV_495	NA	NA	NA	NA	NA	NA	NA	NA	NA
ASV_330	NA	NA	NA	NA	NA	NA	NA	NA	NA
ASV_315	2.858622	0.424135	0.471079	0.448329	0.368205	0.417241	0.231032	0.160434	3.093318
ASV_19	3.866011	0.304009	0.561919	0.308817	0.902564	0.448664	0.212404	0.177473	2.31541
ASV_36	7.587141	0.72812	0.354798	0.597131	1.218244	0.601995	0.238299	0.093549	1.337701
ASV_402	NA	NA	NA	NA	NA	NA	NA	NA	NA
ASV_482	NA	NA	NA	NA	NA	NA	NA	NA	NA

ASVID	BL/BD	EH/BD	ED/HD	MO/BD	JL/HD	HD/BD	PFI/BD	PFL/BL	CFD/CPD
ASV_358	2.55644	0.238141	0.465291	0.189325	0.569434	0.413895	0.135149	0.175961	2.517773
ASV_2	4.395304	0.430567	0.261956	0.388756	0.327939	0.499823	0.24652	0.151566	1.686316
ASV_168	3.570692	0.613874	0.378915	0.562646	0.156111	0.613992	0.376594	0.205325	1.929961
ASV_241	NA	NA	NA	NA	NA	NA	NA	NA	NA
ASV_387	5.167303	0.860773	0.281463	0.491529	0.668039	0.916246	0.670245	0.24011	1.557659
ASV_294	NA	NA	NA	NA	NA	NA	NA	NA	NA
ASV_119	NA	NA	NA	NA	NA	NA	NA	NA	NA
ASV_150	3.967475	0.523949	0.332815	0.464346	0.6455	0.575299	0.147277	0.169048	2.559573
ASV_553	3.512324	0.462105	0.389313	0.462177	0.302473	0.472876	0.220223	0.173139	2.069789
ASV_439	5.19801	0.578772	0.235414	0.563543	0.567453	0.604142	0.375665	0.107194	2.141052
ASV_58	NA	NA	NA	NA	NA	NA	NA	NA	NA
ASV_335	NA	NA	NA	NA	NA	NA	NA	NA	NA
ASV_260	4.238629	0.536093	0.397459	0.453398	0.28108	0.496269	0.263406	0.172173	2.244378
ASV_653	3.808511	0.617031	0.594481	0.509134	0.345738	0.462921	0.489707	0.19787	2.111884
ASV_251	7.587141	0.72812	0.354798	0.597131	1.218244	0.601995	0.238299	0.093549	1.337701
ASV_72	NA	NA	NA	NA	NA	NA	NA	NA	NA
ASV_139	NA	NA	NA	NA	NA	NA	NA	NA	NA
ASV_377	4.238629	0.536093	0.397459	0.453398	0.28108	0.496269	0.263406	0.172173	2.244378
ASV_227	NA	NA	NA	NA	NA	NA	NA	NA	NA
ASV_389	7.375111	0.570125	0.679456	0.460222	1.619584	0.3475	0.541847	0.118703	2.51403
ASV_316	NA	NA	NA	NA	NA	NA	NA	NA	NA
ASV_67	4.698873	0.799085	0.288624	0.446362	0.344732	0.87743	0.753847	0.268948	1.521564
ASV_276	4.506638	0.742256	0.172539	0.544557	0.64476	0.862734	0.79948	0.211437	1.933169
ASV_683	3.570692	0.613874	0.378915	0.562646	0.156111	0.613992	0.376594	0.205325	1.929961
ASV_209	4.200283	0.533449	0.608816	0.644464	0.435943	0.383369	0.388887	0.200773	2.613008
ASV_352	NA	NA	NA	NA	NA	NA	NA	NA	NA
ASV_231	3.808511	0.617031	0.594481	0.509134	0.345738	0.462921	0.489707	0.19787	2.111884
ASV_288	4.825942	0.845115	0.391378	0.708737	0.330631	0.837906	0.579433	0.247546	1.714348
ASV_404	NA	NA	NA	NA	NA	NA	NA	NA	NA
ASV_486	NA	NA	NA	NA	NA	NA	NA	NA	NA

ASVID	BL/BD	EH/BD	ED/HD	MO/BD	JL/HD	HD/BD	PFI/BD	PFL/BL	CFD/CPD
ASV_307	NA	NA	NA	NA	NA	NA	NA	NA	NA
ASV_107	NA	NA	NA	NA	NA	NA	NA	NA	NA
ASV_532	NA	NA	NA	NA	NA	NA	NA	NA	NA
ASV_332	5.19801	0.578772	0.235414	0.563543	0.567453	0.604142	0.375665	0.107194	2.141052
ASV_171	4.200283	0.533449	0.608816	0.644464	0.435943	0.383369	0.388887	0.200773	2.613008
ASV_63	NA	NA	NA	NA	NA	NA	NA	NA	NA
ASV_266	4.238629	0.536093	0.397459	0.453398	0.28108	0.496269	0.263406	0.172173	2.244378
ASV_52	NA	NA	NA	NA	NA	NA	NA	NA	NA
ASV_24	NA	NA	NA	NA	NA	NA	NA	NA	NA
ASV_134	NA	NA	NA	NA	NA	NA	NA	NA	NA
ASV_338	NA	NA	NA	NA	NA	NA	NA	NA	NA
ASV_621	NA	NA	NA	NA	NA	NA	NA	NA	NA
ASV_638	NA	NA	NA	NA	NA	NA	NA	NA	NA
ASV_164	NA	NA	NA	NA	NA	NA	NA	NA	NA
ASV_664	4.825942	0.845115	0.391378	0.708737	0.330631	0.837906	0.579433	0.247546	1.714348
ASV_94	5.19801	0.578772	0.235414	0.563543	0.567453	0.604142	0.375665	0.107194	2.141052
ASV_104	2.204373	0.474314	0.333152	0.427894	0.360825	0.503246	0.255912	0.158555	3.05464
ASV_64	NA	NA	NA	NA	NA	NA	NA	NA	NA
ASV_87	NA	NA	NA	NA	NA	NA	NA	NA	NA
ASV_57	NA	NA	NA	NA	NA	NA	NA	NA	NA
ASV_388	2.858622	0.424135	0.471079	0.448329	0.368205	0.417241	0.231032	0.160434	3.093318
ASV_9	6.146028	0.594757	0.256454	0.379636	0.744375	0.763486	0.26005	0.153479	1.301101
ASV_62	4.522242	0.725575	0.333082	0.579094	0.667965	0.841509	0.821319	0.20109	1.856155
ASV_70	NA	NA	NA	NA	NA	NA	NA	NA	NA
ASV_398	3.212466	0.427381	0.417938	0.577176	0.24111	0.599592	0.221485	0.24947	2.08706
ASV_188	4.286795	0.390298	0.439129	0.22287	0.337223	0.587638	0.20144	0.183425	2.140562
ASV_180	5.167303	0.860773	0.281463	0.491529	0.668039	0.916246	0.670245	0.24011	1.557659
ASV_284	NA	NA	NA	NA	NA	NA	NA	NA	NA
ASV_146	4.716911	0.562914	0.352426	0.251571	0.673874	0.713826	0.160374	0.177559	3.0899
ASV_322	NA	NA	NA	NA	NA	NA	NA	NA	NA

ASVID	BL/BD	EH/BD	ED/HD	MO/BD	JL/HD	HD/BD	PFI/BD	PFL/BL	CFD/CPD
ASV_5	4.825942	0.845115	0.391378	0.708737	0.330631	0.837906	0.579433	0.247546	1.714348
ASV_591	4.200283	0.533449	0.608816	0.644464	0.435943	0.383369	0.388887	0.200773	2.613008
ASV_44	NA	NA	NA	NA	NA	NA	NA	NA	NA
ASV_78	3.570692	0.613874	0.378915	0.562646	0.156111	0.613992	0.376594	0.205325	1.929961
ASV_40	3.570692	0.613874	0.378915	0.562646	0.156111	0.613992	0.376594	0.205325	1.929961
ASV_292	4.716911	0.562914	0.352426	0.251571	0.673874	0.713826	0.160374	0.177559	3.0899
ASV_259	3.570692	0.613874	0.378915	0.562646	0.156111	0.613992	0.376594	0.205325	1.929961
ASV_372	NA	NA	NA	NA	NA	NA	NA	NA	NA
ASV_35	2.858622	0.424135	0.471079	0.448329	0.368205	0.417241	0.231032	0.160434	3.093318
ASV_634	NA	NA	NA	NA	NA	NA	NA	NA	NA
ASV_301	NA	NA	NA	NA	NA	NA	NA	NA	NA
ASV_102	4.506638	0.742256	0.172539	0.544557	0.64476	0.862734	0.79948	0.211437	1.933169
ASV_175	NA	NA	NA	NA	NA	NA	NA	NA	NA
ASV_336	NA	NA	NA	NA	NA	NA	NA	NA	NA
ASV_356	2.858622	0.424135	0.471079	0.448329	0.368205	0.417241	0.231032	0.160434	3.093318
ASV_341	2.514532	0.323241	0.320339	0.251525	0.378519	0.514526	0.20105	0.192218	3.088346
ASV_600	NA	NA	NA	NA	NA	NA	NA	NA	NA
ASV_580	NA	NA	NA	NA	NA	NA	NA	NA	NA
ASV_253	NA	NA	NA	NA	NA	NA	NA	NA	NA
ASV_224	4.642457	0.989068	0.295348	1.017942	0.673488	0.8383	0.675189	0.20749	1.274122
ASV_49	NA	NA	NA	NA	NA	NA	NA	NA	NA
ASV_12	NA	NA	NA	NA	NA	NA	NA	NA	NA
ASV_106	5.167303	0.860773	0.281463	0.491529	0.668039	0.916246	0.670245	0.24011	1.557659
ASV_434	NA	NA	NA	NA	NA	NA	NA	NA	NA
ASV_219	NA	NA	NA	NA	NA	NA	NA	NA	NA
ASV_148	3.212466	0.427381	0.417938	0.577176	0.24111	0.599592	0.221485	0.24947	2.08706
ASV_420	NA	NA	NA	NA	NA	NA	NA	NA	NA
ASV_514	3.212466	0.427381	0.417938	0.577176	0.24111	0.599592	0.221485	0.24947	2.08706
ASV_156	3.512324	0.462105	0.389313	0.462177	0.302473	0.472876	0.220223	0.173139	2.069789
ASV_558	NA	NA	NA	NA	NA	NA	NA	NA	NA

ASVID	BL/BD	EH/BD	ED/HD	MO/BD	JL/HD	HD/BD	PFI/BD	PFL/BL	CFD/CPD
ASV_89	5.19801	0.578772	0.235414	0.563543	0.567453	0.604142	0.375665	0.107194	2.141052
ASV_165	4.200283	0.533449	0.608816	0.644464	0.435943	0.383369	0.388887	0.200773	2.613008
ASV_132	NA	NA	NA	NA	NA	NA	NA	NA	NA
ASV_238	4.286795	0.390298	0.439129	0.22287	0.337223	0.587638	0.20144	0.183425	2.140562
ASV_314	NA	NA	NA	NA	NA	NA	NA	NA	NA
ASV_100	NA	NA	NA	NA	NA	NA	NA	NA	NA
ASV_138	NA	NA	NA	NA	NA	NA	NA	NA	NA
ASV_28	4.378869	0.499995	0.233188	0.472139	0.402741	0.676057	0.279118	0.207759	2.030789
ASV_557	NA	NA	NA	NA	NA	NA	NA	NA	NA
ASV_562	2.416672	0.500026	0.177206	0.281443	0.393066	0.588563	0.296875	0.204752	2.44289
ASV_25	5.167303	0.860773	0.281463	0.491529	0.668039	0.916246	0.670245	0.24011	1.557659
ASV_160	5.167303	0.860773	0.281463	0.491529	0.668039	0.916246	0.670245	0.24011	1.557659
ASV_526	2.623816	0.315941	0.474016	0.35104	0.251575	0.257103	0.166621	0.174364	2.582142
ASV_232	4.522242	0.725575	0.333082	0.579094	0.667965	0.841509	0.821319	0.20109	1.856155
ASV_351	4.200283	0.533449	0.608816	0.644464	0.435943	0.383369	0.388887	0.200773	2.613008
ASV_61	NA	NA	NA	NA	NA	NA	NA	NA	NA
ASV_27	4.716911	0.562914	0.352426	0.251571	0.673874	0.713826	0.160374	0.177559	3.0899
ASV_54	7.375111	0.570125	0.679456	0.460222	1.619584	0.3475	0.541847	0.118703	2.51403
ASV_193	4.716911	0.562914	0.352426	0.251571	0.673874	0.713826	0.160374	0.177559	3.0899
ASV_184	NA	NA	NA	NA	NA	NA	NA	NA	NA
ASV_131	3.212466	0.427381	0.417938	0.577176	0.24111	0.599592	0.221485	0.24947	2.08706
ASV_277	3.570692	0.613874	0.378915	0.562646	0.156111	0.613992	0.376594	0.205325	1.929961
ASV_312	3.512324	0.462105	0.389313	0.462177	0.302473	0.472876	0.220223	0.173139	2.069789
ASV_516	4.413266	0.438557	0.243201	0.438557	0.807943	0.578451	0.240771	0.187279	1.367709
ASV_111	NA	NA	NA	NA	NA	NA	NA	NA	NA
ASV_465	3.512324	0.462105	0.389313	0.462177	0.302473	0.472876	0.220223	0.173139	2.069789
ASV_572	4.200283	0.533449	0.608816	0.644464	0.435943	0.383369	0.388887	0.200773	2.613008
ASV_225	4.825942	0.845115	0.391378	0.708737	0.330631	0.837906	0.579433	0.247546	1.714348
ASV_367	13.42302	0.752358	0.532967	0.495411	4.128165	0.849087	0.472997	0.098307	3.339305
ASV_122	3.212466	0.427381	0.417938	0.577176	0.24111	0.599592	0.221485	0.24947	2.08706

ASVID	BL/BD	EH/BD	ED/HD	MO/BD	JL/HD	HD/BD	PFI/BD	PFL/BL	CFD/CPD
ASV_229	3.512324	0.462105	0.389313	0.462177	0.302473	0.472876	0.220223	0.173139	2.069789
ASV_123	NA	NA	NA	NA	NA	NA	NA	NA	NA
ASV_121	5.167303	0.860773	0.281463	0.491529	0.668039	0.916246	0.670245	0.24011	1.557659
ASV_419	3.512324	0.462105	0.389313	0.462177	0.302473	0.472876	0.220223	0.173139	2.069789
ASV_306	NA	NA	NA	NA	NA	NA	NA	NA	NA
ASV_281	3.570692	0.613874	0.378915	0.562646	0.156111	0.613992	0.376594	0.205325	1.929961
ASV_484	4.506638	0.742256	0.172539	0.544557	0.64476	0.862734	0.79948	0.211437	1.933169
ASV_596	NA	NA	NA	NA	NA	NA	NA	NA	NA
ASV_50	NA	NA	NA	NA	NA	NA	NA	NA	NA
ASV_449	4.506638	0.742256	0.172539	0.544557	0.64476	0.862734	0.79948	0.211437	1.933169
ASV_21	NA	NA	NA	NA	NA	NA	NA	NA	NA
ASV_409	4.038552	0.491119	0.279741	0.483013	0.348289	0.684303	0.19161	0.175852	2.273856
ASV_6	NA	NA	NA	NA	NA	NA	NA	NA	NA
ASV_203	4.200283	0.533449	0.608816	0.644464	0.435943	0.383369	0.388887	0.200773	2.613008
ASV_222	4.200283	0.533449	0.608816	0.644464	0.435943	0.383369	0.388887	0.200773	2.613008
ASV_99	4.506638	0.742256	0.172539	0.544557	0.64476	0.862734	0.79948	0.211437	1.933169
ASV_162	NA	NA	NA	NA	NA	NA	NA	NA	NA
ASV_305	3.212466	0.427381	0.417938	0.577176	0.24111	0.599592	0.221485	0.24947	2.08706
ASV_297	NA	NA	NA	NA	NA	NA	NA	NA	NA
ASV_632	3.212466	0.427381	0.417938	0.577176	0.24111	0.599592	0.221485	0.24947	2.08706
ASV_23	NA	NA	NA	NA	NA	NA	NA	NA	NA
ASV_17	4.506638	0.742256	0.172539	0.544557	0.64476	0.862734	0.79948	0.211437	1.933169
ASV_408	NA	NA	NA	NA	NA	NA	NA	NA	NA
ASV_187	5.167303	0.860773	0.281463	0.491529	0.668039	0.916246	0.670245	0.24011	1.557659
ASV_249	4.542123	0.677769	0.425633	0.432227	0.502071	0.634437	0.279669	0.189343	2.474074
ASV_348	3.212466	0.427381	0.417938	0.577176	0.24111	0.599592	0.221485	0.24947	2.08706
ASV_334	4.716911	0.562914	0.352426	0.251571	0.673874	0.713826	0.160374	0.177559	3.0899
ASV_353	NA	NA	NA	NA	NA	NA	NA	NA	NA

Table S2. Morphological traits (unitless ratios) of each ASV after interpolation.

ASVID	BL/BD	EH/BD	ED/HD	MO/BD	JL/HD	HD/BD	PFI/BD	PFL/BL	CFD/CPD
ASV_71	2.85144	0.428981	0.347788	0.370802	0.419502	0.511079	0.244433	0.170662	2.912992
ASV_468	3.212466	0.427381	0.417938	0.577176	0.24111	0.599592	0.221485	0.24947	2.08706
ASV_103	5.830272	0.65186	0.437305	0.502688	1.057632	0.632397	0.55324	0.174404	2.222621
ASV_405	4.825942	0.845115	0.391378	0.708737	0.330631	0.837906	0.579433	0.247546	1.714348
ASV_265	2.523926	0.450171	0.390155	0.431296	0.368802	0.469144	0.244878	0.160345	3.058221
ASV_120	5.491451	0.649809	0.402386	0.500644	0.903512	0.653754	0.537802	0.179259	2.141744
ASV_248	4.542123	0.677769	0.425633	0.432227	0.502071	0.634437	0.279669	0.189343	2.474074
ASV_47	4.179464	0.574374	0.690359	0.651595	0.47403	0.39095	0.307862	0.227394	2.030046
ASV_480	4.201465	0.515869	0.378367	0.440701	0.567364	0.576396	0.247792	0.186921	2.191458
ASV_455	4.825942	0.845115	0.391378	0.708737	0.330631	0.837906	0.579433	0.247546	1.714348
ASV_239	5.789508	0.655339	0.427197	0.504165	1.047257	0.639021	0.568889	0.17518	2.21235
ASV_18	4.183808	0.535713	0.6105	0.641135	0.437797	0.388452	0.378774	0.203137	2.552193
ASV_320	3.392954	0.481176	0.413342	0.471936	0.255132	0.479143	0.275784	0.193427	2.223754
ASV_515	3.866011	0.304009	0.561919	0.308817	0.902564	0.448664	0.212404	0.177473	2.31541
ASV_395	4.780516	0.556182	0.32112	0.416008	0.621067	0.618134	0.273439	0.163849	2.116527
ASV_280	3.570692	0.613874	0.378915	0.562646	0.156111	0.613992	0.376594	0.205325	1.929961
ASV_448	4.103606	0.527707	0.578993	0.61926	0.428948	0.412616	0.363661	0.202827	2.509189
ASV_201	4.996371	0.886617	0.301258	0.647111	0.624211	0.883687	0.657884	0.232773	1.517772
ASV_535	4.506638	0.742256	0.172539	0.544557	0.64476	0.862734	0.79948	0.211437	1.933169
ASV_475	4.698873	0.799085	0.288624	0.446362	0.344732	0.87743	0.753847	0.268948	1.521564
ASV_10	3.845877	0.502829	0.435474	0.513486	0.418519	0.532277	0.281065	0.199969	2.272055
ASV_452	2.523926	0.450171	0.390155	0.431296	0.368802	0.469144	0.244878	0.160345	3.058221
ASV_144	3.570692	0.613874	0.378915	0.562646	0.156111	0.613992	0.376594	0.205325	1.929961
ASV_533	4.506638	0.742256	0.172539	0.544557	0.64476	0.862734	0.79948	0.211437	1.933169
ASV_129	2.815345	0.512375	0.39286	0.529604	0.348707	0.418344	0.314064	0.204297	2.491324
ASV_59	4.201465	0.515869	0.378367	0.440701	0.567364	0.576396	0.247792	0.186921	2.191458
ASV_654	3.212466	0.427381	0.417938	0.577176	0.24111	0.599592	0.221485	0.24947	2.08706
ASV_546	4.200283	0.533449	0.608816	0.644464	0.435943	0.383369	0.388887	0.200773	2.613008
ASV_65	4.551415	0.409171	0.491778	0.425822	0.993017	0.456379	0.284047	0.200356	1.615996

ASVID	BL/BD	EH/BD	ED/HD	MO/BD	JL/HD	HD/BD	PFI/BD	PFL/BL	CFD/CPD
ASV_118	4.506638	0.742256	0.172539	0.544557	0.64476	0.862734	0.79948	0.211437	1.933169
ASV_649	5.463383	0.658746	0.397731	0.502507	0.88391	0.664464	0.544912	0.182606	2.114127
ASV_101	4.506638	0.742256	0.172539	0.544557	0.64476	0.862734	0.79948	0.211437	1.933169
ASV_115	2.858622	0.424135	0.471079	0.448329	0.368205	0.417241	0.231032	0.160434	3.093318
ASV_417	3.710242	0.524258	0.255189	0.362992	0.255635	0.758452	0.209831	0.202143	3.345455
ASV_230	2.874666	0.505389	0.218225	0.301296	0.432583	0.595177	0.280029	0.198304	2.43712
ASV_507	7.027284	0.588675	0.624522	0.469823	1.493553	0.411546	0.546118	0.131167	2.448131
ASV_243	3.512324	0.462105	0.389313	0.462177	0.302473	0.472876	0.220223	0.173139	2.069789
ASV_206	2.760167	0.504048	0.20797	0.296333	0.422703	0.593523	0.284241	0.199916	2.438562
ASV_429	4.200283	0.533449	0.608816	0.644464	0.435943	0.383369	0.388887	0.200773	2.613008
ASV_13	4.845633	0.565202	0.325781	0.437439	0.654941	0.598404	0.299903	0.157057	2.099933
ASV_254	4.103606	0.527707	0.578993	0.61926	0.428948	0.412616	0.363661	0.202827	2.509189
ASV_69	4.493937	0.65894	0.420537	0.434928	0.506418	0.627402	0.275645	0.189129	2.44638
ASV_354	5.240592	0.641702	0.483148	0.504568	0.849996	0.582966	0.53471	0.181248	2.190322
ASV_555	5.463383	0.658746	0.397731	0.502507	0.88391	0.664464	0.544912	0.182606	2.114127
ASV_550	5.624455	0.734304	0.138965	0.491324	0.596095	0.583831	0.398839	0.120422	1.141527
ASV_43	13.42302	0.752358	0.532967	0.495411	4.128165	0.849087	0.472997	0.098307	3.339305
ASV_304	3.512324	0.462105	0.389313	0.462177	0.302473	0.472876	0.220223	0.173139	2.069789
ASV_393	4.716911	0.562914	0.352426	0.251571	0.673874	0.713826	0.160374	0.177559	3.0899
ASV_299	5.830272	0.65186	0.437305	0.502688	1.057632	0.632397	0.55324	0.174404	2.222621
ASV_166	3.570692	0.613874	0.378915	0.562646	0.156111	0.613992	0.376594	0.205325	1.929961
ASV_437	4.698873	0.799085	0.288624	0.446362	0.344732	0.87743	0.753847	0.268948	1.521564
ASV_55	5.615017	0.727153	0.149164	0.49008	0.605492	0.584918	0.392488	0.1217	1.183507
ASV_423	5.491451	0.649809	0.402386	0.500644	0.903512	0.653754	0.537802	0.179259	2.141744
ASV_339	4.61518	0.71831	0.339728	0.571571	0.690552	0.823505	0.794133	0.198997	1.88354
ASV_127	2.204373	0.474314	0.333152	0.427894	0.360825	0.503246	0.255912	0.158555	3.05464
ASV_416	5.491451	0.649809	0.402386	0.500644	0.903512	0.653754	0.537802	0.179259	2.141744
ASV_142	4.825942	0.845115	0.391378	0.708737	0.330631	0.837906	0.579433	0.247546	1.714348
ASV_397	5.167303	0.860773	0.281463	0.491529	0.668039	0.916246	0.670245	0.24011	1.557659
ASV_359	7.587141	0.72812	0.354798	0.597131	1.218244	0.601995	0.238299	0.093549	1.337701

ASVID	BL/BD	EH/BD	ED/HD	MO/BD	JL/HD	HD/BD	PFI/BD	PFL/BL	CFD/CPD
ASV_68	4.642457	0.989068	0.295348	1.017942	0.673488	0.8383	0.675189	0.20749	1.274122
ASV_114	5.463383	0.658746	0.397731	0.502507	0.88391	0.664464	0.544912	0.182606	2.114127
ASV_606	3.845877	0.502829	0.435474	0.513486	0.418519	0.532277	0.281065	0.199969	2.272055
ASV_383	4.47438	0.453794	0.273394	0.403595	0.384736	0.516187	0.266762	0.151354	1.77372
ASV_214	5.443378	0.553784	0.575095	0.552946	0.543931	0.40663	0.639276	0.174314	2.281855
ASV_46	4.47438	0.453794	0.273394	0.403595	0.384736	0.516187	0.266762	0.151354	1.77372
ASV_415	7.176353	0.580725	0.648065	0.465708	1.547567	0.384098	0.544288	0.125825	2.476373
ASV_672	5.830272	0.65186	0.437305	0.502688	1.057632	0.632397	0.55324	0.174404	2.222621
ASV_155	4.47438	0.453794	0.273394	0.403595	0.384736	0.516187	0.266762	0.151354	1.77372
ASV_41	5.789508	0.655339	0.427197	0.504165	1.047257	0.639021	0.568889	0.17518	2.21235
ASV_124	3.136575	0.426098	0.433567	0.431638	0.253946	0.40513	0.239396	0.187073	2.343217
ASV_252	5.624455	0.734304	0.138965	0.491324	0.596095	0.583831	0.398839	0.120422	1.141527
ASV_178	4.156632	0.527138	0.384863	0.453836	0.536844	0.578156	0.247473	0.187632	2.252525
ASV_189	5.491451	0.649809	0.402386	0.500644	0.903512	0.653754	0.537802	0.179259	2.141744
ASV_303	4.200283	0.533449	0.608816	0.644464	0.435943	0.383369	0.388887	0.200773	2.613008
ASV_378	5.240592	0.641702	0.483148	0.504568	0.849996	0.582966	0.53471	0.181248	2.190322
ASV_563	4.716911	0.562914	0.352426	0.251571	0.673874	0.713826	0.160374	0.177559	3.0899
ASV_30	5.189713	0.578052	0.237011	0.561513	0.568102	0.603637	0.375038	0.108044	2.140362
ASV_495	4.201465	0.515869	0.378367	0.440701	0.567364	0.576396	0.247792	0.186921	2.191458
ASV_330	5.789508	0.655339	0.427197	0.504165	1.047257	0.639021	0.568889	0.17518	2.21235
ASV_315	2.858622	0.424135	0.471079	0.448329	0.368205	0.417241	0.231032	0.160434	3.093318
ASV_19	3.866011	0.304009	0.561919	0.308817	0.902564	0.448664	0.212404	0.177473	2.31541
ASV_36	7.587141	0.72812	0.354798	0.597131	1.218244	0.601995	0.238299	0.093549	1.337701
ASV_402	5.789508	0.655339	0.427197	0.504165	1.047257	0.639021	0.568889	0.17518	2.21235
ASV_482	3.845877	0.502829	0.435474	0.513486	0.418519	0.532277	0.281065	0.199969	2.272055
ASV_358	2.55644	0.238141	0.465291	0.189325	0.569434	0.413895	0.135149	0.175961	2.517773
ASV_2	4.395304	0.430567	0.261956	0.388756	0.327939	0.499823	0.24652	0.151566	1.686316
ASV_168	3.570692	0.613874	0.378915	0.562646	0.156111	0.613992	0.376594	0.205325	1.929961
ASV_241	5.491451	0.649809	0.402386	0.500644	0.903512	0.653754	0.537802	0.179259	2.141744
ASV_387	5.167303	0.860773	0.281463	0.491529	0.668039	0.916246	0.670245	0.24011	1.557659

ASVID	BL/BD	EH/BD	ED/HD	MO/BD	JL/HD	HD/BD	PFI/BD	PFL/BL	CFD/CPD
ASV_294	7.300577	0.5741	0.667685	0.46228	1.592578	0.361224	0.542762	0.121373	2.499909
ASV_119	5.240592	0.641702	0.483148	0.504568	0.849996	0.582966	0.53471	0.181248	2.190322
ASV_150	3.967475	0.523949	0.332815	0.464346	0.6455	0.575299	0.147277	0.169048	2.559573
ASV_553	3.512324	0.462105	0.389313	0.462177	0.302473	0.472876	0.220223	0.173139	2.069789
ASV_439	5.19801	0.578772	0.235414	0.563543	0.567453	0.604142	0.375665	0.107194	2.141052
ASV_58	5.463383	0.658746	0.397731	0.502507	0.88391	0.664464	0.544912	0.182606	2.114127
ASV_335	5.491451	0.649809	0.402386	0.500644	0.903512	0.653754	0.537802	0.179259	2.141744
ASV_260	4.238629	0.536093	0.397459	0.453398	0.28108	0.496269	0.263406	0.172173	2.244378
ASV_653	3.808511	0.617031	0.594481	0.509134	0.345738	0.462921	0.489707	0.19787	2.111884
ASV_251	7.587141	0.72812	0.354798	0.597131	1.218244	0.601995	0.238299	0.093549	1.337701
ASV_72	5.491451	0.649809	0.402386	0.500644	0.903512	0.653754	0.537802	0.179259	2.141744
ASV_139	2.760167	0.504048	0.20797	0.296333	0.422703	0.593523	0.284241	0.199916	2.438562
ASV_377	4.238629	0.536093	0.397459	0.453398	0.28108	0.496269	0.263406	0.172173	2.244378
ASV_227	3.614211	0.441573	0.413697	0.391701	0.538538	0.491751	0.259623	0.163547	2.571029
ASV_389	7.375111	0.570125	0.679456	0.460222	1.619584	0.3475	0.541847	0.118703	2.51403
ASV_316	4.183808	0.535713	0.6105	0.641135	0.437797	0.388452	0.378774	0.203137	2.552193
ASV_67	4.698873	0.799085	0.288624	0.446362	0.344732	0.87743	0.753847	0.268948	1.521564
ASV_276	4.506638	0.742256	0.172539	0.544557	0.64476	0.862734	0.79948	0.211437	1.933169
ASV_683	3.570692	0.613874	0.378915	0.562646	0.156111	0.613992	0.376594	0.205325	1.929961
ASV_209	4.200283	0.533449	0.608816	0.644464	0.435943	0.383369	0.388887	0.200773	2.613008
ASV_352	4.017062	0.507438	0.340754	0.413487	0.56227	0.559957	0.290296	0.163271	2.448504
ASV_231	3.808511	0.617031	0.594481	0.509134	0.345738	0.462921	0.489707	0.19787	2.111884
ASV_288	4.825942	0.845115	0.391378	0.708737	0.330631	0.837906	0.579433	0.247546	1.714348
ASV_404	2.760167	0.504048	0.20797	0.296333	0.422703	0.593523	0.284241	0.199916	2.438562
ASV_486	2.85864	0.364462	0.461499	0.354444	0.457992	0.42606	0.202883	0.166025	2.831831
ASV_307	3.845877	0.502829	0.435474	0.513486	0.418519	0.532277	0.281065	0.199969	2.272055
ASV_107	5.830272	0.65186	0.437305	0.502688	1.057632	0.632397	0.55324	0.174404	2.222621
ASV_532	5.830272	0.65186	0.437305	0.502688	1.057632	0.632397	0.55324	0.174404	2.222621
ASV_332	5.19801	0.578772	0.235414	0.563543	0.567453	0.604142	0.375665	0.107194	2.141052
ASV_171	4.200283	0.533449	0.608816	0.644464	0.435943	0.383369	0.388887	0.200773	2.613008

ASVID	BL/BD	EH/BD	ED/HD	MO/BD	JL/HD	HD/BD	PFI/BD	PFL/BL	CFD/CPD
ASV_63	5.615017	0.727153	0.149164	0.49008	0.605492	0.584918	0.392488	0.1217	1.183507
ASV_266	4.238629	0.536093	0.397459	0.453398	0.28108	0.496269	0.263406	0.172173	2.244378
ASV_52	4.017062	0.507438	0.340754	0.413487	0.56227	0.559957	0.290296	0.163271	2.448504
ASV_24	5.830272	0.65186	0.437305	0.502688	1.057632	0.632397	0.55324	0.174404	2.222621
ASV_134	4.183808	0.535713	0.6105	0.641135	0.437797	0.388452	0.378774	0.203137	2.552193
ASV_338	5.830272	0.65186	0.437305	0.502688	1.057632	0.632397	0.55324	0.174404	2.222621
ASV_621	7.226042	0.578075	0.655913	0.464337	1.565571	0.374948	0.543677	0.124044	2.485788
ASV_638	6.479666	0.671416	0.327212	0.531267	0.971046	0.601589	0.269174	0.118343	1.586842
ASV_164	2.523926	0.450171	0.390155	0.431296	0.368802	0.469144	0.244878	0.160345	3.058221
ASV_664	4.825942	0.845115	0.391378	0.708737	0.330631	0.837906	0.579433	0.247546	1.714348
ASV_94	5.19801	0.578772	0.235414	0.563543	0.567453	0.604142	0.375665	0.107194	2.141052
ASV_104	2.204373	0.474314	0.333152	0.427894	0.360825	0.503246	0.255912	0.158555	3.05464
ASV_64	3.136575	0.426098	0.433567	0.431638	0.253946	0.40513	0.239396	0.187073	2.343217
ASV_87	4.201465	0.515869	0.378367	0.440701	0.567364	0.576396	0.247792	0.186921	2.191458
ASV_57	4.493937	0.65894	0.420537	0.434928	0.506418	0.627402	0.275645	0.189129	2.44638
ASV_388	2.858622	0.424135	0.471079	0.448329	0.368205	0.417241	0.231032	0.160434	3.093318
ASV_9	6.146028	0.594757	0.256454	0.379636	0.744375	0.763486	0.26005	0.153479	1.301101
ASV_62	4.522242	0.725575	0.333082	0.579094	0.667965	0.841509	0.821319	0.20109	1.856155
ASV_70	3.392954	0.481176	0.413342	0.471936	0.255132	0.479143	0.275784	0.193427	2.223754
ASV_398	3.212466	0.427381	0.417938	0.577176	0.24111	0.599592	0.221485	0.24947	2.08706
ASV_188	4.286795	0.390298	0.439129	0.22287	0.337223	0.587638	0.20144	0.183425	2.140562
ASV_180	5.167303	0.860773	0.281463	0.491529	0.668039	0.916246	0.670245	0.24011	1.557659
ASV_284	5.491451	0.649809	0.402386	0.500644	0.903512	0.653754	0.537802	0.179259	2.141744
ASV_146	4.716911	0.562914	0.352426	0.251571	0.673874	0.713826	0.160374	0.177559	3.0899
ASV_322	4.50601	0.463085	0.277969	0.40953	0.407455	0.522733	0.274858	0.151269	1.808682
ASV_5	4.825942	0.845115	0.391378	0.708737	0.330631	0.837906	0.579433	0.247546	1.714348
ASV_591	4.200283	0.533449	0.608816	0.644464	0.435943	0.383369	0.388887	0.200773	2.613008
ASV_44	3.845877	0.502829	0.435474	0.513486	0.418519	0.532277	0.281065	0.199969	2.272055
ASV_78	3.570692	0.613874	0.378915	0.562646	0.156111	0.613992	0.376594	0.205325	1.929961
ASV_40	3.570692	0.613874	0.378915	0.562646	0.156111	0.613992	0.376594	0.205325	1.929961

ASVID	BL/BD	EH/BD	ED/HD	MO/BD	JL/HD	HD/BD	PFI/BD	PFL/BL	CFD/CPD
ASV_292	4.716911	0.562914	0.352426	0.251571	0.673874	0.713826	0.160374	0.177559	3.0899
ASV_259	3.570692	0.613874	0.378915	0.562646	0.156111	0.613992	0.376594	0.205325	1.929961
ASV_372	5.491451	0.649809	0.402386	0.500644	0.903512	0.653754	0.537802	0.179259	2.141744
ASV_35	2.858622	0.424135	0.471079	0.448329	0.368205	0.417241	0.231032	0.160434	3.093318
ASV_634	5.491451	0.649809	0.402386	0.500644	0.903512	0.653754	0.537802	0.179259	2.141744
ASV_301	5.240592	0.641702	0.483148	0.504568	0.849996	0.582966	0.53471	0.181248	2.190322
ASV_102	4.506638	0.742256	0.172539	0.544557	0.64476	0.862734	0.79948	0.211437	1.933169
ASV_175	5.789508	0.655339	0.427197	0.504165	1.047257	0.639021	0.568889	0.17518	2.21235
ASV_336	3.103663	0.50807	0.238735	0.311223	0.452341	0.598484	0.271606	0.19508	2.434234
ASV_356	2.858622	0.424135	0.471079	0.448329	0.368205	0.417241	0.231032	0.160434	3.093318
ASV_341	2.514532	0.323241	0.320339	0.251525	0.378519	0.514526	0.20105	0.192218	3.088346
ASV_600	5.830272	0.65186	0.437305	0.502688	1.057632	0.632397	0.55324	0.174404	2.222621
ASV_580	5.463383	0.658746	0.397731	0.502507	0.88391	0.664464	0.544912	0.182606	2.114127
ASV_253	5.491451	0.649809	0.402386	0.500644	0.903512	0.653754	0.537802	0.179259	2.141744
ASV_224	4.642457	0.989068	0.295348	1.017942	0.673488	0.8383	0.675189	0.20749	1.274122
ASV_49	3.219065	0.428398	0.418201	0.576008	0.243298	0.598329	0.222582	0.248719	2.089175
ASV_12	2.760167	0.504048	0.20797	0.296333	0.422703	0.593523	0.284241	0.199916	2.438562
ASV_106	5.167303	0.860773	0.281463	0.491529	0.668039	0.916246	0.670245	0.24011	1.557659
ASV_434	7.428931	0.720019	0.350857	0.587722	1.18293	0.601937	0.24271	0.097091	1.373292
ASV_219	4.014101	0.518125	0.313153	0.492187	0.329529	0.614607	0.295644	0.20377	2.06754
ASV_148	3.212466	0.427381	0.417938	0.577176	0.24111	0.599592	0.221485	0.24947	2.08706
ASV_420	5.491451	0.649809	0.402386	0.500644	0.903512	0.653754	0.537802	0.179259	2.141744
ASV_514	3.212466	0.427381	0.417938	0.577176	0.24111	0.599592	0.221485	0.24947	2.08706
ASV_156	3.512324	0.462105	0.389313	0.462177	0.302473	0.472876	0.220223	0.173139	2.069789
ASV_558	5.830272	0.65186	0.437305	0.502688	1.057632	0.632397	0.55324	0.174404	2.222621
ASV_89	5.19801	0.578772	0.235414	0.563543	0.567453	0.604142	0.375665	0.107194	2.141052
ASV_165	4.200283	0.533449	0.608816	0.644464	0.435943	0.383369	0.388887	0.200773	2.613008
ASV_132	3.991179	0.517786	0.537545	0.588984	0.417768	0.446528	0.340139	0.203202	2.431579
ASV_238	4.286795	0.390298	0.439129	0.22287	0.337223	0.587638	0.20144	0.183425	2.140562
ASV_314	5.463383	0.658746	0.397731	0.502507	0.88391	0.664464	0.544912	0.182606	2.114127

ASVID	BL/BD	EH/BD	ED/HD	MO/BD	JL/HD	HD/BD	PFI/BD	PFL/BL	CFD/CPD
ASV_100	5.615017	0.727153	0.149164	0.49008	0.605492	0.584918	0.392488	0.1217	1.183507
ASV_138	3.845877	0.502829	0.435474	0.513486	0.418519	0.532277	0.281065	0.199969	2.272055
ASV_28	4.378869	0.499995	0.233188	0.472139	0.402741	0.676057	0.279118	0.207759	2.030789
ASV_557	5.240592	0.641702	0.483148	0.504568	0.849996	0.582966	0.53471	0.181248	2.190322
ASV_562	2.416672	0.500026	0.177206	0.281443	0.393066	0.588563	0.296875	0.204752	2.44289
ASV_25	5.167303	0.860773	0.281463	0.491529	0.668039	0.916246	0.670245	0.24011	1.557659
ASV_160	5.167303	0.860773	0.281463	0.491529	0.668039	0.916246	0.670245	0.24011	1.557659
ASV_526	2.623816	0.315941	0.474016	0.35104	0.251575	0.257103	0.166621	0.174364	2.582142
ASV_232	4.522242	0.725575	0.333082	0.579094	0.667965	0.841509	0.821319	0.20109	1.856155
ASV_351	4.200283	0.533449	0.608816	0.644464	0.435943	0.383369	0.388887	0.200773	2.613008
ASV_61	5.830272	0.65186	0.437305	0.502688	1.057632	0.632397	0.55324	0.174404	2.222621
ASV_27	4.716911	0.562914	0.352426	0.251571	0.673874	0.713826	0.160374	0.177559	3.0899
ASV_54	7.375111	0.570125	0.679456	0.460222	1.619584	0.3475	0.541847	0.118703	2.51403
ASV_193	4.716911	0.562914	0.352426	0.251571	0.673874	0.713826	0.160374	0.177559	3.0899
ASV_184	4.47438	0.453794	0.273394	0.403595	0.384736	0.516187	0.266762	0.151354	1.77372
ASV_131	3.212466	0.427381	0.417938	0.577176	0.24111	0.599592	0.221485	0.24947	2.08706
ASV_277	3.570692	0.613874	0.378915	0.562646	0.156111	0.613992	0.376594	0.205325	1.929961
ASV_312	3.512324	0.462105	0.389313	0.462177	0.302473	0.472876	0.220223	0.173139	2.069789
ASV_516	4.413266	0.438557	0.243201	0.438557	0.807943	0.578451	0.240771	0.187279	1.367709
ASV_111	3.766324	0.497946	0.454648	0.528431	0.395408	0.514353	0.293096	0.203951	2.276359
ASV_465	3.512324	0.462105	0.389313	0.462177	0.302473	0.472876	0.220223	0.173139	2.069789
ASV_572	4.200283	0.533449	0.608816	0.644464	0.435943	0.383369	0.388887	0.200773	2.613008
ASV_225	4.825942	0.845115	0.391378	0.708737	0.330631	0.837906	0.579433	0.247546	1.714348
ASV_367	13.42302	0.752358	0.532967	0.495411	4.128165	0.849087	0.472997	0.098307	3.339305
ASV_122	3.212466	0.427381	0.417938	0.577176	0.24111	0.599592	0.221485	0.24947	2.08706
ASV_229	3.512324	0.462105	0.389313	0.462177	0.302473	0.472876	0.220223	0.173139	2.069789
ASV_123	5.789508	0.655339	0.427197	0.504165	1.047257	0.639021	0.568889	0.17518	2.21235
ASV_121	5.167303	0.860773	0.281463	0.491529	0.668039	0.916246	0.670245	0.24011	1.557659
ASV_419	3.512324	0.462105	0.389313	0.462177	0.302473	0.472876	0.220223	0.173139	2.069789
ASV_306	4.014101	0.518125	0.313153	0.492187	0.329529	0.614607	0.295644	0.20377	2.06754

ASVID	BL/BD	EH/BD	ED/HD	MO/BD	JL/HD	HD/BD	PFI/BD	PFL/BL	CFD/CPD
ASV_281	3.570692	0.613874	0.378915	0.562646	0.156111	0.613992	0.376594	0.205325	1.929961
ASV_484	4.506638	0.742256	0.172539	0.544557	0.64476	0.862734	0.79948	0.211437	1.933169
ASV_596	3.991179	0.517786	0.537545	0.588984	0.417768	0.446528	0.340139	0.203202	2.431579
ASV_50	5.240592	0.641702	0.483148	0.504568	0.849996	0.582966	0.53471	0.181248	2.190322
ASV_449	4.506638	0.742256	0.172539	0.544557	0.64476	0.862734	0.79948	0.211437	1.933169
ASV_21	3.520542	0.462773	0.388694	0.46108	0.305339	0.47448	0.22044	0.173214	2.073147
ASV_409	4.038552	0.491119	0.279741	0.483013	0.348289	0.684303	0.19161	0.175852	2.273856
ASV_6	5.491451	0.649809	0.402386	0.500644	0.903512	0.653754	0.537802	0.179259	2.141744
ASV_203	4.200283	0.533449	0.608816	0.644464	0.435943	0.383369	0.388887	0.200773	2.613008
ASV_222	4.200283	0.533449	0.608816	0.644464	0.435943	0.383369	0.388887	0.200773	2.613008
ASV_99	4.506638	0.742256	0.172539	0.544557	0.64476	0.862734	0.79948	0.211437	1.933169
ASV_162	2.934197	0.372173	0.456719	0.35817	0.466047	0.432629	0.208557	0.165777	2.805751
ASV_305	3.212466	0.427381	0.417938	0.577176	0.24111	0.599592	0.221485	0.24947	2.08706
ASV_297	5.240592	0.641702	0.483148	0.504568	0.849996	0.582966	0.53471	0.181248	2.190322
ASV_632	3.212466	0.427381	0.417938	0.577176	0.24111	0.599592	0.221485	0.24947	2.08706
ASV_23	2.523926	0.450171	0.390155	0.431296	0.368802	0.469144	0.244878	0.160345	3.058221
ASV_17	4.506638	0.742256	0.172539	0.544557	0.64476	0.862734	0.79948	0.211437	1.933169
ASV_408	2.760167	0.504048	0.20797	0.296333	0.422703	0.593523	0.284241	0.199916	2.438562
ASV_187	5.167303	0.860773	0.281463	0.491529	0.668039	0.916246	0.670245	0.24011	1.557659
ASV_249	4.542123	0.677769	0.425633	0.432227	0.502071	0.634437	0.279669	0.189343	2.474074
ASV_348	3.212466	0.427381	0.417938	0.577176	0.24111	0.599592	0.221485	0.24947	2.08706
ASV_334	4.716911	0.562914	0.352426	0.251571	0.673874	0.713826	0.160374	0.177559	3.0899
ASV_353	5.830272	0.65186	0.437305	0.502688	1.057632	0.632397	0.55324	0.174404	2.222621

Table S3. Generalized linear regression (GLM) fitting results of taxonomic diversity, functional diversity, and genetic diversity of fishes in the Yuan River Basin in relation to elevation and human footprint, as well as the effects of spatial autocorrelation and tributary. Numbers are coefficients with standard errors (SE) in parentheses, and more asterisks indicate that the response of the corresponding parameter to the corresponding diversity is more significant (i.e. '***' means $p<0.001$, '**' means $p<0.01$, '*' means $p<0.05$, and no asterisk means $p>0.05$). Autocor is a spatial autocovariate by extracting the GLM model residuals and then using it as a spatial variable independent of the factors considered in the study to predict fish biodiversity facets. The colon represents the interaction. Note that the elevation, taxonomic diversity, functional diversity and genetic diversity variables were scaled before running.

Parameters	taxonomic diversity	functional diversity	genetic diversity
(Intercept)	2.1725 (0.5334)***	1.6339 (0.7003)*	-0.1803 (0.6495)
elevation	-0.3975 (0.1734)*	-0.2567 (0.2276)	0.4345 (0.2111)*
footprint	-0.0799 (0.0231)**	-0.0644 (0.0303)*	-0.0016 (0.0281)
autocor	-0.4477 (0.4542)	-0.4035 (0.5963)	0.2093 (0.5519)
tributary	-1.1756 (0.3344)**	-0.7338 (0.4391)	0.3857 (0.4073)
elevation:footprint	n.s.	n.s.	n.s.

Table S4. The AIC values of direct model (mod0) and random models (GLM_Random). All parameters were detailed in **Text S4**.

Facet	Factor	AIC_mod0	AIC_GLM_Random
alpha_taxonomic	Elevation	77.6076	90.53985
alpha_function	Elevation	95.57862	105.7937
alpha_genetic	Elevation	90.61414	98.31145
alpha_taxonomic	Footprint	77.6076	90.07287
alpha_function	Footprint	95.57862	105.7937
alpha_genetic	Footprint	90.61414	101.5858

Supplementary Figures

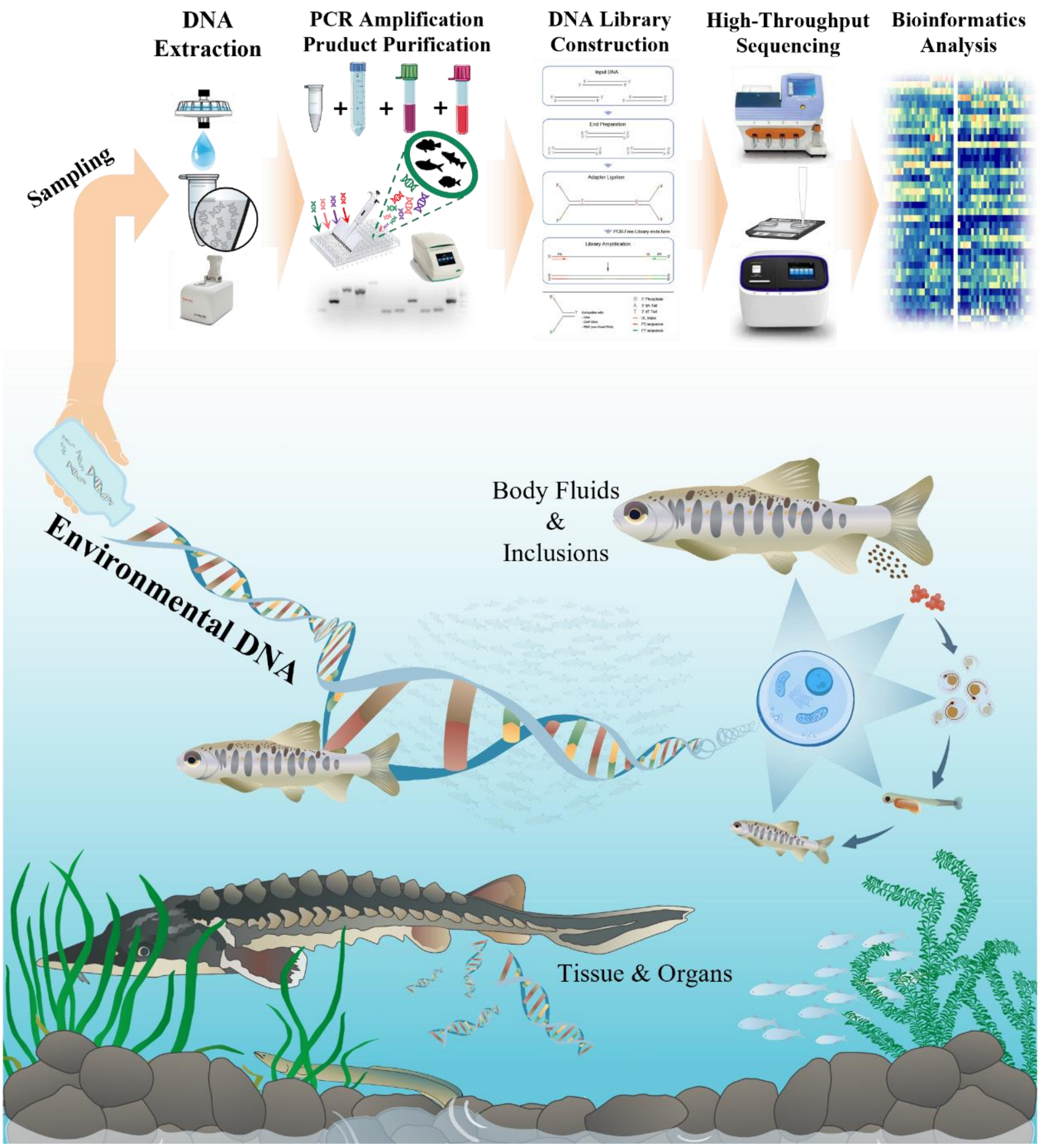

Figure S1. Sources of environmental DNA (eDNA) from fish in rivers and standard operating procedures (SOPs) for fish monitoring using eDNA. All symbols used in this figure were downloaded from https://ian.umces.edu/media-library and phylopic.org.

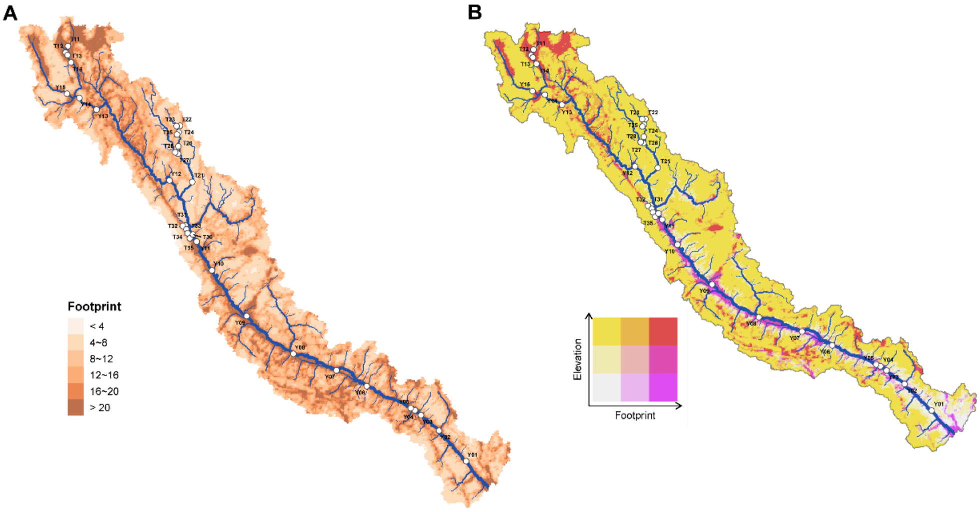

Figure S2. Human footprint distribution (A) and its joint distribution pattern with elevation (B) in the Yuan River Basin.

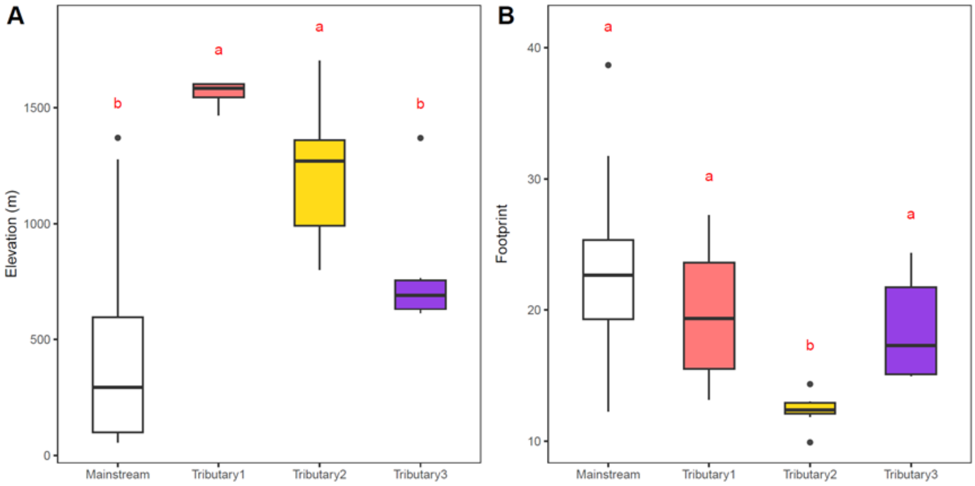

Figure S3. Wilcox tests of elevation and footprint among three tributaries and mainstream.

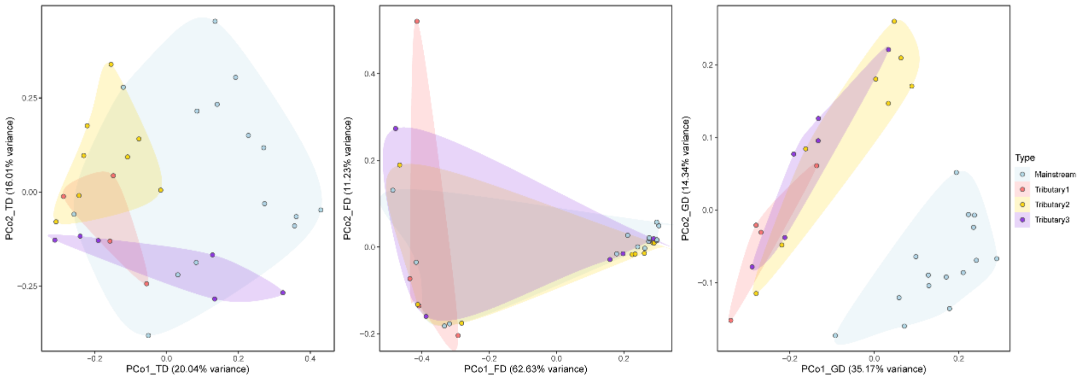

Figure S4. PCoA analyses of taxonomic, functional and genetic diversity among three tributaries and mainstream.

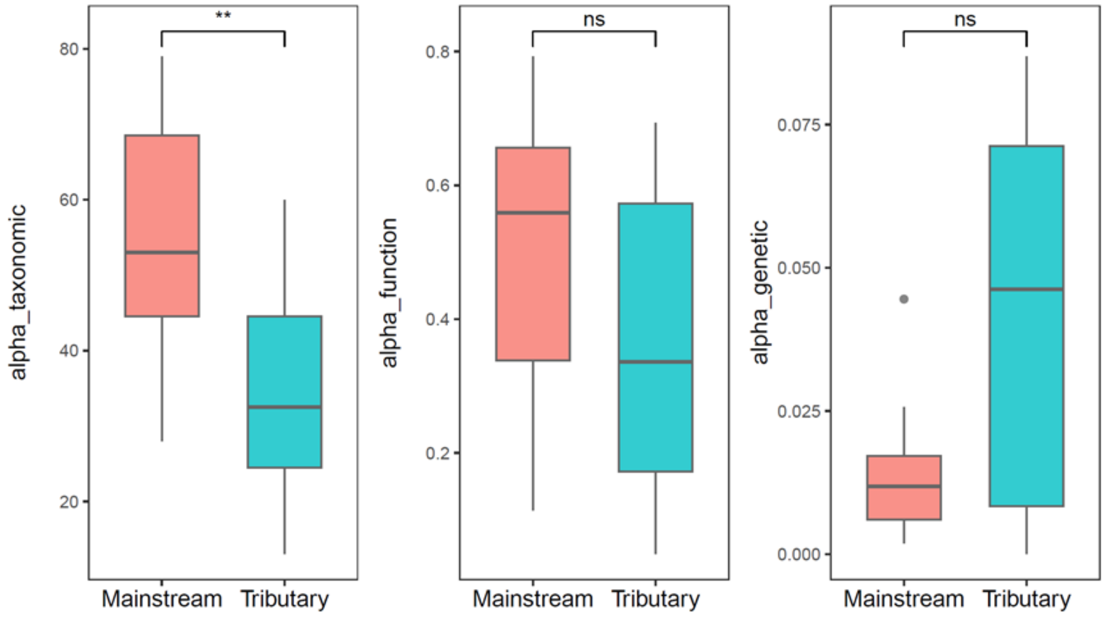

Figure S5. Kruskal-Wallis tests of taxonomic, functional and genetic diversity between mainstream and tributary.

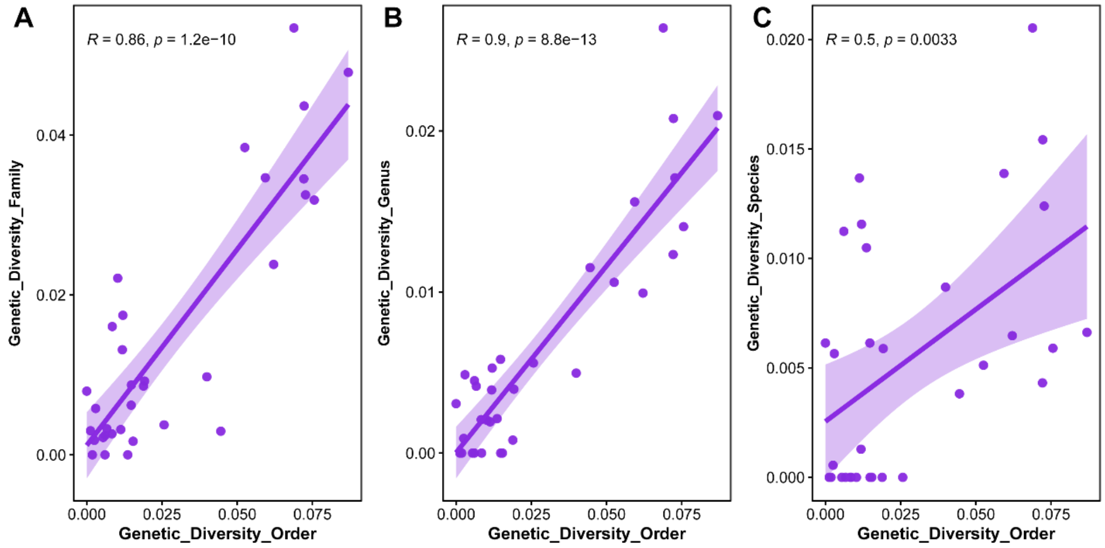

Figure S6. Congruence between genetic diversity at the order level and genetic diversity at the family (A), genus (B) or species (C) level, respectively. The solid line is the significant relationship found by the linear model (LM) with 95% confidence interval.

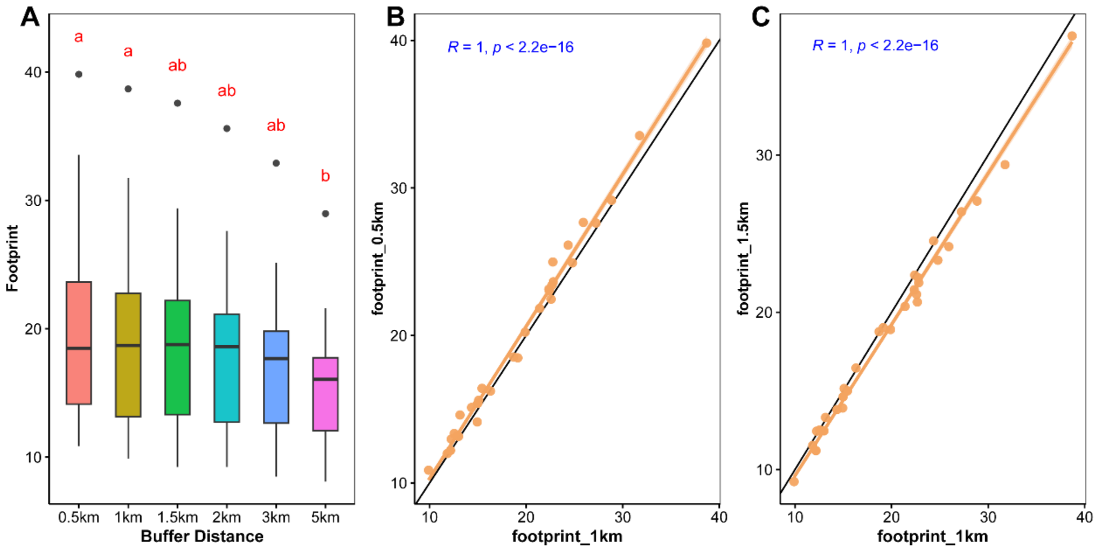

Figure S7. Comparisons of buffer distances. (A) Wilcoxon tests of footprint values among different buffer distance; (B) Correlation between the values of 1 km buffer and 0.5 km buffer; (C) Correlation between the values of 1 km buffer and 1.5 km buffer.

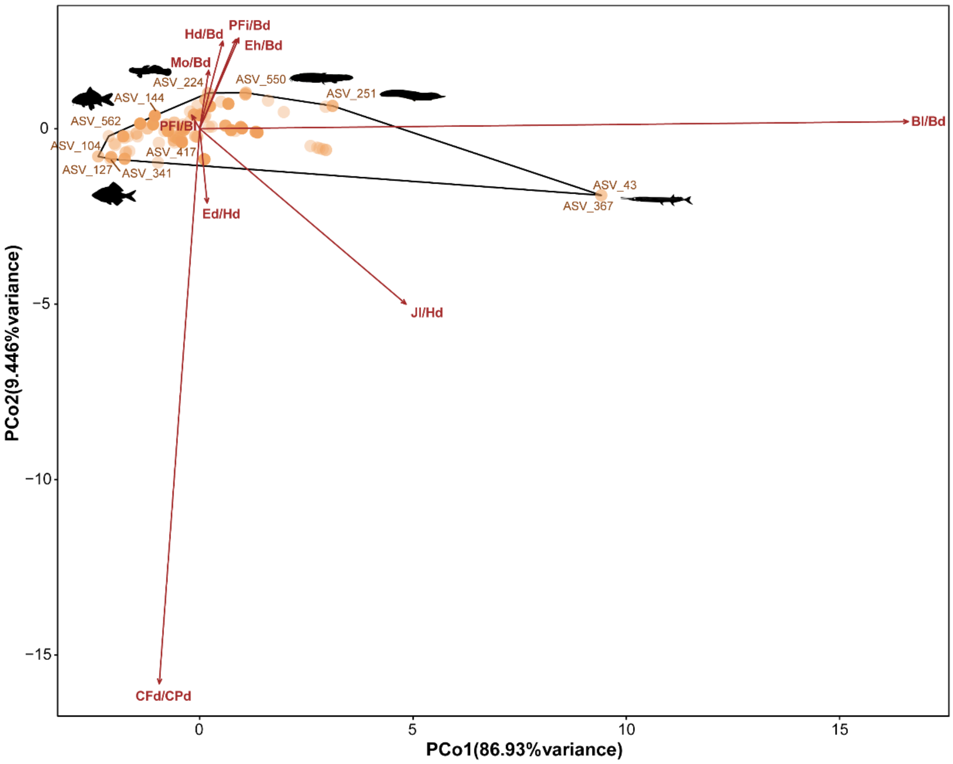

Figure S8. Functional space and trait loadings on the first two principal axes based on principal coordinate analysis of ASVs of Yuan River fish. Each trait is shown in bold to indicate that the trait is significant and highly correlated with the first principal axis or the second principal axis, and the longer length of the line segment represents the higher loading of the trait. Only the species corresponding to the outermost ASVs of the functional space are silhouetted.

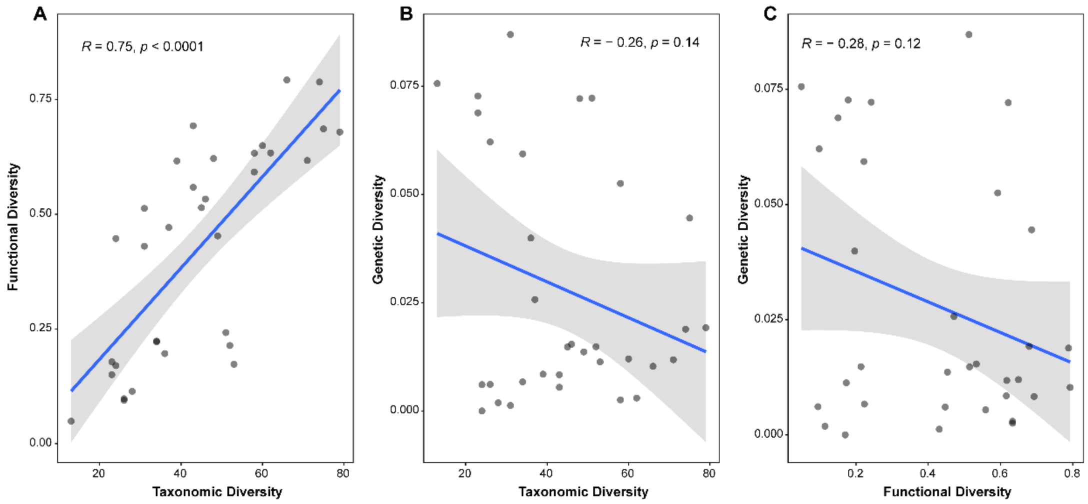

Figure S9. Relationships of fish "taxonomic diversity - functional diversity" (A), "taxonomic diversity - genetic diversity" (B) and "functional diversity - genetic diversity" (C) in the Yuan River. The solid line is the significant relationship found by generalized linear model (GLM) with 95% confidence interval.

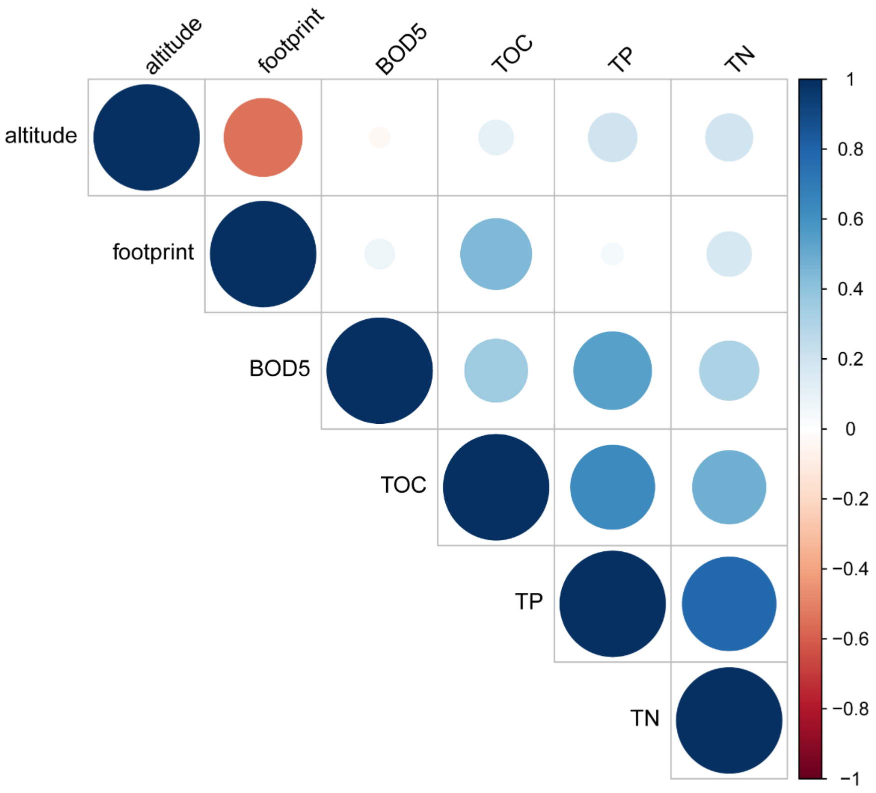

Figure S10. Co-correlation of environmental factors in the Yuan River. The size of the circle indicates significance, i.e. the larger the circle, the more significant the paired factor. The colour from dark to light indicates the correlation, the darker the colour the stronger the correlation, and the correlation is divided into two directions: positive and negative.
